# Viral Mimicry as a Design Template for Nucleic Acid Nanocarriers

**DOI:** 10.3389/fchem.2021.613209

**Published:** 2021-03-10

**Authors:** Ina F. de la Fuente, Shraddha S. Sawant, Mark Q. Tolentino, Patrick M. Corrigan, Jessica L. Rouge

**Affiliations:** Department of Chemistry, University of Connecticut, Storrs, CT, United States

**Keywords:** nucleic acid delivery, viral mimicry, endosomal escape, nuclear targeting, nanoparticles

## Abstract

Therapeutic nucleic acids hold immense potential in combating undruggable, gene-based diseases owing to their high programmability and relative ease of synthesis. While the delivery of this class of therapeutics has successfully entered the clinical setting, extrahepatic targeting, endosomal escape efficiency, and subcellular localization remain as major roadblocks. On the other hand, viruses serve as natural carriers of nucleic acids and have acquired a plethora of structures and mechanisms that confer remarkable transfection efficiency. Thus, understanding the structure and mechanism of viruses can guide the design of synthetic nucleic acid vectors. This review revisits relevant structural and mechanistic features of viruses as design considerations for efficient nucleic acid delivery systems. This article explores how viral ligand display and a metastable structure are central to the molecular mechanisms of attachment, entry, and viral genome release. For comparison, accounted for are details on the design and intracellular fate of existing nucleic acid carriers and nanostructures that share similar and essential features to viruses. The review, thus, highlights unifying themes of viruses and nucleic acid delivery systems such as genome protection, target specificity, and controlled release. Sophisticated viral mechanisms that are yet to be exploited in oligonucleotide delivery are also identified as they could further the development of next-generation nonviral nucleic acid vectors.

## Introduction

Undruggable targets are disease-implicated proteins that lack easy-to-bind pockets where conventional therapeutics like small molecules can bind (Crews, 2010; Duffy and Crown, 2021). However, around 80% of the human proteome is difficult to reach or target (Verdine and Walensky, 2007). The past decade has shown enormous progress in targeting the previously thought to be unreachable sites such as growth factors, enzymes, defective genes, or nuclear transcription factors (Lazo and Sharlow, 2016). In particular, therapeutic nucleic acids such as small interfering RNAs (siRNAs), microRNAs (miRNAs), antisense oligonucleotides (ASOs), synthetic messenger RNAs (mRNAs), and CRISPR-Cas9-guide RNAs are programmable, easy to synthesize, and thus have the potential to treat previously undruggable diseases such as cancer and viral diseases (Dowdy, 2017). They hold great promise in treating the root cause of the disease rather than just treating the symptoms by targeting the mutated genes, mRNA, or proteins with high specificity and selectivity (Keefe et al., 2010; Damha 2019). The challenge lies in delivery (Juliano, 2016; Dowdy, 2017; Dowdy and Levy, 2018; Johannes and Lucchino, 2018; Juliano, 2018).

For billions of years, cells have evolved to keep genomic material on one side of the membrane. Thus, transfection by bare nucleic acids across an anionic lipid barrier is fundamentally prevented by the large size and density of negative charges (Dowdy, 2017; Dowdy and Levy, 2018; Johannes and Lucchino, 2018). Furthermore, medical translation necessitates a successful *in vivo* delivery. This is particularly challenging given the limited systemic stability of unmodified nucleic acids. Thus, an ideal delivery strategy should include nucleic acid protection from nuclease degradation and oxidation, prolonged systemic circulation, targeted delivery, efficient transfection across a membrane, facilitated access to the cytoplasm or nucleus, and little to no side effects (Zhu and Mahato, 2010). While progress has been made in designing and implementing safe, effective, and efficient nucleic acid delivery systems, realizing their therapeutic potential is, at present, challenged mainly by the lack of cellular target diversity and endosomal escape ability (Dowdy, 2017; Dowdy and Levy, 2018; Johannes and Lucchino, 2018; Juliano, 2018).

**FIGURE 1:**
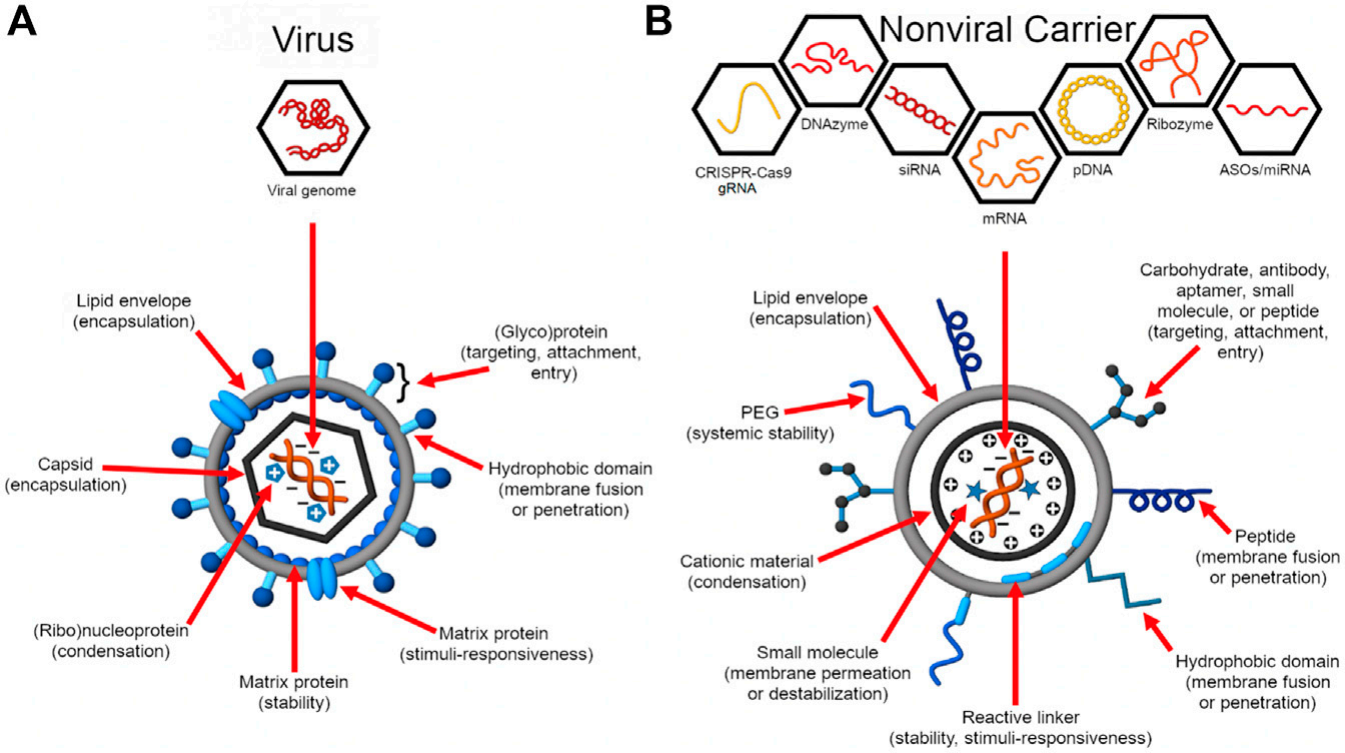
Virus structure and function inform the design of nucleic acid delivery systems. **(A)** Viruses evolve to deliver their genome efficiently to the host cell for replication (Flint et al., 2015). As such, their genome encodes proteins essential for genome protection, tropism, intracellular trafficking, controlled genome release, and replication. **(B)** Synthetic carriers are designed to deliver a diversity of therapeutic nucleic acid cargo including pDNA, siRNA, ASOs, miRNA, mRNA, CRISPR-Cas9 guide RNAs (gRNAs), ribozymes, and DNAzymes (Ni et al., 2016; Ni et al., 2019). Analogous to viruses, functional domains are embedded on the construct that enable a balance between nucleic acid protection and programmed, stimulus-induced release.

In contrast, viruses have evolved a diversity of enabling architectures for the infiltration of various host cells and controlled viral genome replication using the host cell machinery (Flint et al., 2015). While they have become longstanding models for engineering the transfection of therapeutic nucleic acids (Figure 1) (Ni et al., 2016), their delivery efficiency far outplays that of synthetic vectors (Ramamoorth and Narvekar, 2015). This underscores how our current molecular understanding of viral function and how this relates to nucleic acid transfection can be improved to achieve more effective translation to rational design.

This review, therefore, details the structure and intracellular fate of existing nucleic acid delivery strategies whose designs are either directly inspired by viruses or their resulting formulation exhibits many similarities to that of viruses. Hence, relevant structural and mechanistic features of viruses as design considerations for viable nucleic acid delivery systems are examined. This article also explores how a dynamic and stimulus-responsive structure can play an important role in designing an effective nucleic acid carrier. Importantly, it also highlights how sophisticated ligand display is central to the molecular mechanisms of carrier trafficking and nucleic acid release.

## General Structure of Nucleic Acid Carriers and Mechanism of Protection

**FIGURE 2:**
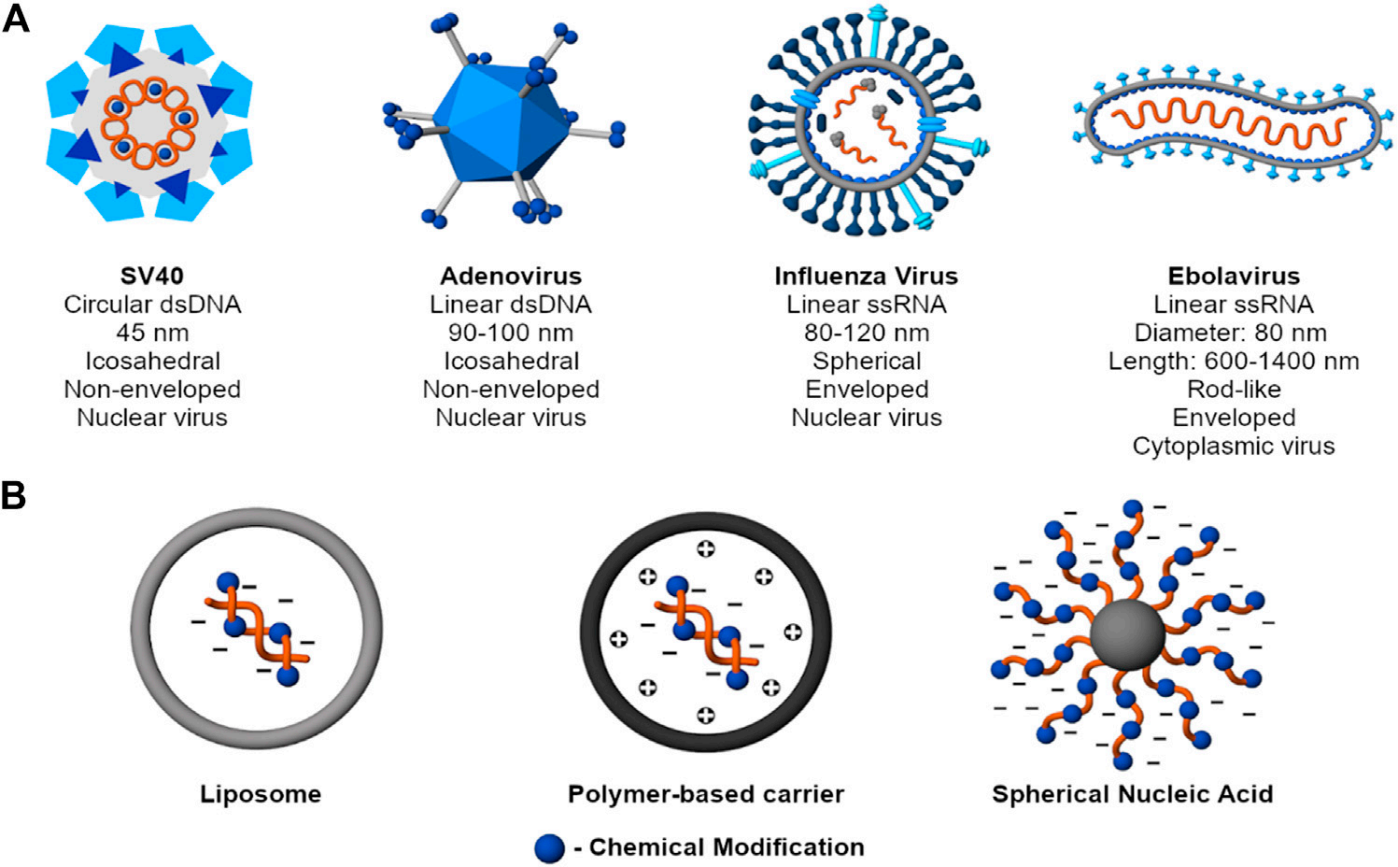
Mechanisms to protect nucleic acid cargo. **(A)** Examples of common viruses (SV40 - Martini et al. 2007; Adenovirus - Greber et al., 1997; Russel, 2009; Influenza Virus - James and Whitley, 2017; Ebolavirus - Beniac et al., 2012; Falasca et al., 2015). Despite structural diversity, viruses collectively protect their genome through charge condensation and encapsulation by a capsid and, for an enveloped virus, an outer lipid membrane. **(B)** Examples of nonviral nucleic acid delivery systems. Beyond condensation and encapsulation, nonviral carriers also use chemical modifications, self-generated sterics, or a combination of strategies to achieve the same purpose.

An ideal carrier packs, stores, and protects nucleic acid cargo until it has reached the target site. In that regard, this section provides examples of select viruses and nonviral nucleic acid vectors and discusses their structural features relevant to the efficient packing and protection of nucleic acids. Figure 2 presents examples of common viruses to show that despite differences in sizes and shapes, viruses collectively protect their genome through condensation and encapsulation. In addition to these two mechanisms of nucleic acid protection, nonviral carriers also use chemical modifications, self-generated sterics, or a combination of these strategies to achieve the same effect.

### Structure of Viruses and Genome Protection

Viruses are obligate intracellular parasites (Gelderblom, 1996). They have evolved to transfect their DNA or RNA genome into the host cell for expression and subsequent production of more virus particles (Prasad and Schmid, 2011). At the core of virus structure are structural proteins that serve to protect the viral genome until it is delivered to the target site. These structural proteins assemble to form the viral capsid, which is the protein coat that wraps around the genome. The high degree of folding and dense packing of capsid proteins protect them from proteolytic digestion, making them stable carriers of nucleic acid cargo (Flint et al., 2015). Moreover, the viral genome is typically condensed by viral proteins through charge neutralization (Gelderblom, 1996), allowing confinement within the interior of the capsid. Enveloped viruses possess an outer lipid envelope that provides additional encapsulation and can fuse with the host plasma membrane during uptake or endosomal escape. The protein components encoded by the viral genome display highly specific and often, multiple, roles essential for structural integrity, attachment, and replication in the host cell (Flint et al., 2015).

For example, the main components of the influenza virus are the lipid bilayer, glycoprotein spikes hemagglutinin (HA) and neuraminidase (NA), matrix proteins (M1 and M2), the heterotrimeric RNA-dependent RNA polymerase (RdRP), the viral RNA segments, a nucleoprotein (NP), and two nonstructural proteins (NS1 and NS2 a.k.a. nuclear export protein or NEP). The outermost layer of the virus is a lipid membrane decorated with glycoproteins that, in turn, may be recognized by antibodies to protect the host against infection (James and Whitley, 2017). Thus, these glycoproteins are critical in both immune response and the development of therapeutics. Hemagglutinin, specifically its subunit HA1, is responsible for the targeting of and uptake by the host cells. HA1 binds to sialic acid functionalized cell surface receptors, resulting in receptor-mediated endocytosis. The lipid bilayer is stabilized by M1 on its cytoplasmic periphery and is spanned by M2, a proton ionophore. The core of the virion contains the viral genome as well as proteins essential for viral gene replication (RdRP), gene encapsulation (NP), and nuclear translocation (NEP). Each protein-coding ssRNA segment is coated by NPs and associated with an RdRP, forming a ribonucleoprotein (RNP) complex that is anchored to M1. The viral envelope of influenza virus has been used as a carrier for nucleic acids such as siRNA (de Jonge et al., 2006) and miRNA (Li J. et al., 2013). Particularly, the reconstituted influenza virus membrane envelope, called “virosome,” acts as an efficient carrier to target small nucleic acid such as siRNA *in vitro* as well as *in vivo* (de Jonge et al., 2006). As per this study, the functional integrity of HA viral protein helps in membrane fusion and efficient cytosolic delivery of siRNA.

Another example is the adenovirus (AdV), one of the largest (90–100 nm) non-enveloped double stranded linear DNA viruses. The icosahedral shaped capsid is made of many structural polypeptides. Most of the capsid coat (about 75%) is composed of a hexon protein, which is held together by protein IX. A unique feature of Adv capsid is that the vertices are made of a penton protein from which fiber knobs protrude out–both of which are essential for host cell entry. The viral genome is condensed by proteins V, VII and μ and is also covalently associated with the terminal protein. The cementing protein IIIa acts as capsid stabilizing protein by linking the facets of the icosahedron (Greber et al., 1997; Fay and Panté, 2015). Adenoviral vectors have been used for delivering shRNA, siRNA (Nayerossadat et al., 2012), and large sizes of DNA (up to 38 kb). However, unlike retroviruses, these cannot integrate the carried DNA into the host genome. Thus, the desired gene expression is limited. Also, the immunogenic response caused by adenoviral infection and low cell specificity limits the use of such viral vector only to few tissues such as lungs and liver (Vorburger and Hunt, 2002).

Despite the structural and mechanistic differences among viruses, all viral capsids are metastable, which means they are stable enough to protect the genome until they reach the target site to uncoat it. Thus, the virus construct is spring-loaded in that potential energy is stored during its assembly. Upon reaching the target site, a chemical trigger such as low pH or proteolytic enzymes overcome the energetic barrier, resulting in virus disassembly and uncoating of the genome. Metastability is achieved by the inherent symmetrical arrangement of identical capsid protein subunits that is stabilized by nonspecific noncovalent interactions. In this regard, many capsid proteins self-assemble into virus-like particles (VLPs) (Flint et al., 2015).

VLPs are non-infectious, multiprotein complexes that mimic the viral capsid assembly but are devoid of the genome. Their utility as experimental tools and as therapeutic carriers has been thoroughly reviewed elsewhere (Rohovie et al., 2017; Roldão et al., 2017). Recombinant versions with attenuated or inactivated antigens can also be reconstructed from complementary DNA of a viral genome. While VLPs are historically produced and extracted from the natural hosts themselves, nowadays they are primarily produced through various cell cultures (Roldão et al., 2017). The use of mammalian and non-mammalian cells, baculoviruses, and bacteria has been reported, but VLPs are commonly expressed in yeast cells due to the relative ease of protein expression, scalability, and lower production cost compared to mammalian and insect cells (Kim and Kim, 2017; Roldão et al., 2017).

Like viruses, VLPs have been successfully used in developing vaccines and vaccine adjuvants, and their use in gene therapy and immunotherapy has also been explored (Rohovie et al., 2017; Roldão et al., 2017). Some of those that have shown potential for nucleic acid delivery include bacteriophage-based MS2 (Pan et al., 2012a; Pan et al., 2012b), bacteriophage-based M13 (Yata et al., 2014), animal virus-based hepatitis B virus core (Brandenburg et al., 2005), and plant-based cowpea chlorotic mottle virus (Lam and Steinmetz, 2019).

Target specificity can be tailored by chemical conjugation or directly expressing targeting ligands on the protein coat (Rohovie et al., 2017). For example, Yata et al. (2014) demonstrated the use of a hybrid VLP/cationic polymer-based system for efficient gene transfer. The construct specifically used bacteriophage M13 that was genetically modified to express the RGD peptide on its surface for tumor targeting and was complexed with a cationic polymer for enhanced cellular uptake. Similarly, Lam and Steinmetz (2019) recently delivered siRNA for the knockdown of GFP and FOXA1 target genes using cowpea chlorotic mottle VLPs. With an SM(PEG)_4_ crosslinker, the VLPs were chemically labeled with m-lycotoxin, a cell-penetrating peptide, to enhance cellular uptake.

### Strategies for Nucleic Acid Protection by Nonviral Carriers

While the ability of viruses and VLPs to efficiently encapsulate and transfect nucleic acids is remarkable, they are structurally more complex and, thus, typically require hosts for production and subsequent purification (Roldão et al., 2017), both of which may come at a high cost. Moreover, viruses and VLPs have a higher risk of triggering an immune response (Xue et al., 2015) and possess limited chemistry (Wagner, 2012). Therefore, tuning properties such as target specificity, particle stability, and subcellular localization is restricted, motivating the construction of non-viral vectors (Wagner, 2012). Beyond condensation and encapsulation, this section lists other strategies that have been employed for efficient protection of nucleic acid cargo such as chemical modifications and self-generated sterics. Furthermore, these strategies are often combined for enhanced protection.

#### Condensation by Cationic Materials

Viral assembly mainly involves electrostatic interactions between the capsid proteins and genomic cargo. Similarly, many first-generation designs of delivery agents relied on the electrostatic masking of the polyanionic backbone of nucleic acids for successful delivery into cells. Whereas viruses protect their nucleic acid cargo via capsid encapsulation, cationic materials such as natural and synthetic polymers, dendrimers, proteins, peptides, and cationic lipids as well as inorganic nanoparticles bearing a positive charge (to be discussed in Section *Utility of Inorganic Nanoparticles*) form an electrostatic interaction with the negative phosphate backbone of the nucleic acid cargo, providing protection from nuclease degradation (Ferrari et al., 1999; Moret et al., 2001; Thomas and Klibanov, 2003). This can be ascribed to the compaction of nucleic acids, which results in the blockage of enzymatic digestion sites, thereby conferring nuclease protection (Feng et al., 2015).

Electrostatic interactions also strengthen viral attachment to the surface of negatively—charged host cells. Thus, viruses such as the hepatitis C virus (Penin et al., 2001) and the influenza virus (Arinaminpathy and Grenfell, 2010) have conserved cationic regions in their glycoproteins that aid in membrane binding. In the same light, synthetic polycationic nucleic acid carriers not only allow compaction and protection from nuclease degradation but they also mediate cellular attachment and entry (Mislick and Baldeschwieler, 1996). However, this uptake mechanism is nonspecific, and polymeric materials tend to form aggregates with components of the blood such as serum proteins. For this reason, nonionic, hydrophilic polymers such as PEG are commonly added to confer stealth (Klibanov et al., 1990; Takemoto et al., 2014). Additionally, the structural flexibility of PEG makes its integration into different formulations very convenient. However, while PEG-ylation imparts blood compatibility and circulation longevity (Takemoto et al., 2014), it can compromise cellular uptake and/or endosomal escape (Fang et al., 2017).

To address this limitation, PEG-ylation typically involves responsive linkages that can be cleaved by cellular cues such as low pH or external stimuli such as temperature (Fang et al., 2017). An alternative way of using cleavable PEG was demonstrated by Li and co-workers (2013) where they used MMP-7-cleavable peptides as linkers. Matrix Metalloproteinase-7 (MMP-7) belongs to a class of zinc-dependent, extracellular proteases that are overexpressed on the surface of breast tumor cells. In their construct, the outer surface of the polymer-based siRNA-delivery vector was decorated with PEG attached to the core of the particle using a peptide substrate of MMP-7. When the peptide substrate came to contact with MMP-7, the PEG outer layer was cleaved off, revealing a highly cationic dimethylaminoethyl methacrylate core that then engages the membrane, facilitating uptake. Thus, the selective attachment and entry of the resulting construct is afforded through proximity activation by MMP-7.

Peptide-based vectors tend to rely on positive charge character to condense nucleic acids for packaging and protection. In particular, these consist of cationic amphiphilic peptides that are composed of a hydrophobic and a hydrophilic domain that form a well-defined nanoparticle (Kang et al., 2019). The hydrophobic region consists of non-polar neutral amino acids whereas the hydrophilic region has polar aliphatic residues. These peptides self-assemble to form a micellular structure. Small molecule drugs and DNA can be co-delivered using these multifunctional micelle-plexes, where each peptide plays a different role. For example, displaying a cell penetrating peptide on the surface facilitates binding and entry. Histidine residues cause endosomal escape while lysine residues condense DNA. These types of complexes have been used to deliver siRNA and plasmid DNA. Recent studies have also shown that the addition of stearyl, an alkyl chain, or cholesterol to the hydrophobic domain of self-assembled peptides further enhances DNA condensation and transfection efficiency (Kang et al., 2019).

In addition, highly branched polypeptides are used as hybrid-peptide based gene delivery vehicles. This is achieved by covalently joining multi-functional peptide sequences. Functional peptides are separated by spacers such as repeats of glycine residues that confer flexibility. Nucleic acids are also packed by condensation. Redox-active disulfide bonds can be used to connect peptides in a branched fashion, delivering genes more efficiently than linear counterparts. These disulfide bonds are then reduced in the cytoplasm by glutathione to liberate the nucleic acid cargo as well as to reduce cytotoxicity. Highly branched arginine-rich polypeptides are multivalent and flexible—attributes beneficial for nucleic acid compaction and cellular entry. Many of these reducible multibranched cationic polypeptides have the potential to be non-toxic, degradable vectors for gene delivery (Kang et al., 2019).

Among various polycationic formulations, materials based on synthetic polymers such as polymeric nanoparticles, dendrimers, polymer micelles, polymersomes, polyplexes, and lipopolyplexes have benefited from their chemical diversity, relatively simple design, and potential for multi-functionality (Takemoto et al., 2014; Yuan and Li, 2017). The chemistry, molecular weight, weight relative to the nucleic acid, and overall topology of the polymer determine its stability and transfection efficiency. Intracellularly cleavable linkages are typically inserted within the polymeric chain, affording a dynamic structure that reveals the nucleic acid payload in response to a site-specific stimulus (Troiber and Wagner, 2011).

In a similar sense, multiblock copolymers impart modularity and enable multifunctionality. As an example, polymeric carriers are often based on the electrostatic condensation and shielding by a cationic polymer such as polydimethylaminoethyl methacrylate (pDMAEA). pDMAEA can then be copolymerized with a second block of p(N-(3-(1H-imidazol-1-yl)propyl)acrylamide (pImPAA) and poly(butyl acrylate) (pBA) that mediates an acid-triggered endosomal escape. PImPAA and PBA were designed based on viral membranolytic peptides, and they disrupt the endosomal membrane synergistically through electrostatic and hydrophobic interactions, respectively (Truong et al., 2013; Gillard et al., 2014). Such cationic polymer-based carriers serve as valuable tools for assessing the potency of nucleic acids under study. At this time, structural heterogeneity, imprecise surface conjugation, lack of structure-function insights, and cytotoxicity at therapeutically effective formulations currently hamper their clinical utility (Lv et al., 2006; Troiber and Wagner 2011).

#### Encapsulation by Lipid-Based Vectors

Nucleic acid protection through charge neutralization and condensation by cationic materials may only provide partial nuclease resistance (Moret et al., 2001). Moreover, additional encapsulation by lipid membranes to form lipopolyplexes has been shown to enhance protection from nucleases and the overall therapeutic efficacy of nucleic acids (Yen et al., 2018). For this reason, lipid-based vectors such as liposomes and solid lipid nanoparticles are commonly explored as nucleic acid carriers (Barba et al., 2019). Compared to other nucleic acid delivery systems, lipid-based carriers offer ease of manufacturing and scalability. Their lipid formulation mimics the lipid bilayer, imparting biocompatibility and conveniently facilitating cellular uptake (Ghasemiyeh and Mohammadi-Samani, 2018).

Among these, liposomes have shown the most promise (Barba et al., 2019). They are spherical vesicles made of a lipid bilayer with an aqueous core (Kulkarni et al., 2018; Barba et al., 2019) and can be designed to carry both hydrophilic and lipophilic cargo (Ghasemiyeh and Mohammadi-Samani, 2018; Barba et al., 2019). The earliest work demonstrating liposome-mediated gene delivery was in 1980 by Fraley et al. (1980) when SV40 DNA was encapsulated and delivered using large unilamellar vesicles. They found that using PS exhibited the highest delivery efficiency. Felgner et al. (1987) then showed that using synthetic cationic lipids such as DOTMA resulted in a higher transfection efficiency. Since then, cationic lipids bearing different structure modifications such as DOTAP, DOSPA, DMRIE, and DL-cholesterol have been incorporated in liposome-based gene delivery systems (Zhi et al., 2013; Yin et al., 2014). For anionic cargo such as nucleic acids, the cationic head group permits condensation of the large biomolecule (Zhi et al., 2013). Moreover, polycationic head groups such as polyamines can be used to form polycationic liposomes. These combine the ability of cationic liposomes to complex nucleic acids and that of polycations to mediate endosomal escape via the proton sponge effect (Yamazaki et al., 2000; Sugiyama et al., 2004; Asai et al., 2011; Yonenaga et al., 2012). Nonionic lipids such as fusogenic DOPE and cholesterol can also be incorporated into the liposome to further enhance its stability and delivery efficiency (Wasungu and Hoekstra, 2006).

Modular release usually centers on the lipid formulation where the lipid envelope is destabilized either by an external stimulus such as temperature or an cellular stimulus such as low pH (Heidarli et al., 2017; Aghdam et al., 2019). As an example, Yatvin et al. (1978) introduced the idea that liposomes can preferentially release cargo at the diseased site in response to mild hyperthermic temperature (around 40°C). This was initially achieved using DPPC alone or with DSPC, which has a phase-transition temperature of 42–44°C, above which its membrane permeability increases (Kono et al., 2010; Aghdam et al., 2019). Among efforts that followed on the construction of heat-responsive liposomes (Matsumura and Maeda, 1986; Tomita et al., 1989; Maruyama et al., 1993; Gaber et al., 1995; Anyarambhatla and Needham, 1999; Needham et al., 2000), Anyarambhatla and Needham (1999) notably incorporated a lysolipid to DPPC to bring down the phase-transition temperature to a clinically achievable range (39–40°C) and initiate release within tens of seconds (Needham et al., 2000). As this design only achieved 50% cargo release within an hour at 42°C (Needham et al., 2000), succeeding studies focused on modulating the temperature-responsiveness of liposomes. One strategy is the incorporation of thermosensitive polymers that can impart a sharp and tunable phase transition temperature to the liposome. Upon heating, the polymeric components form hydrophobic domains that disrupt the lipid bilayer (Kono et al., 2010).

On the other hand, pH-sensitive liposomes exploit the differential acidification in the vicinity of malignant tumors or within endosomes for controlled release via membrane fusion or destabilization (Yatvin et al., 1980; Budker et al., 1996; Heidarli et al., 2017). Earlier anionic pH-responsive designs were constructed with a bilayer rich in PE that is stabilized by anionic lipids containing carboxylate head groups at physiological pH (Budker et al., 1996). PE typically forms an inverted hexagonal phase on its own (Chernomordik et al., 1995). Thus, when the anionic carboxylate head groups are protonated in a region of lower pH, the PE-rich bilayer is disrupted (Budker et al., 1996). While there were reports on using anionic liposomes for nucleic acid delivery (Wang and Huang 1989; Legendre and Szoka 1992), their negative charge limits both the efficient packing of polyanionic nucleic acids and interaction with the negatively charged cellular membrane. For this reason, cationic pH-sensitive liposomes were developed. These contain a weakly basic lipid component such as DOTAP and DODAP that have a pKa slightly below physiological pH (Budker et al., 1996; Sato et al., 2012).

Certain early formulations of lipid-based carriers were limited in part by toxicity and immunogenicity at high lipid concentrations, as well as by low bioavailability and low biodistribution (Zatsepin et al., 2016; Huggins et al., 2019). Overtime these formulations have been significantly improved. In addition, the ease of lipid synthesis and structural modifications permit thorough studies on structure-activity relationships and thus, enable a guided design of more efficient and safe delivery systems (Zhi et al., 2013). Furthermore, lipid-based carriers can be easily decorated with receptor ligands to target specific cell types such as tumor and angiogenic endothelial cells (Yonenaga et al., 2012). Such studies culminated in 2018 with the success of Patisiran (ONPATTRO^®^), a liposomal vector developed by Alnylam Pharmaceuticals, as the first US Food and Drug Administration approved synthetic carrier of siRNA into cells (Adams et al., 2018; Hoy, 2018; Wood, 2018).

#### Chemical Modifications

Chemical modifications may impart one or more of the following: *in vivo* stability, cellular delivery, reduced immunogenicity, and potency through enhanced target binding affinity (Judge et al., 2006; Corey, 2007; Whitehead et al., 2009). Such modifications may alter the phosphodiester backbone (phosphothiorates, boranophosphates, and locked nucleic acids), the ribose sugar (2′ modifications, 4′ thio), or the base (ribodifluorotoluyl nucleotide) (Corey 2007). In particular, 2′-O-modifications on siRNA impart nuclease resistance (Whitehead et al., 2009) and suppression of sequence-dependent immunostimulation by some sequences (Judge et al., 2005; Judge et al., 2006). Furthermore, Jackson et al. (Jackson et al., 2006) showed that by specifically modifying position two in the siRNA guide strand, off-target binding of other transcripts to the seed region is reduced. In addition, uncharged nucleic acid mimics such as peptide nucleic acids and morpholino oligomers present unique chemical properties and may improve biodistribution and efficacy. Details on the structure, properties, and applications of chemically modified nucleic acids and DNA/RNA mimics have been extensively reviewed elsewhere (Karkare and Bhatnagar, 2006; Summerton, 2006; Corey, 2007; Chery, 2016).

#### Utility of Inorganic Nanoparticles

Inorganic nanoparticles are emerging as appealing synthetic vectors for nucleic acid delivery owing to their unique properties such as tunable size and surface properties, multifunctional capabilities, chemical and thermal stability, and low inherent toxicity (Loh et al., 2015; Ding et al., 2014a). Incorporating nucleic acid cargo into inorganic nanoparticles can be accomplished using the following general strategies: complexation between negatively charged nucleic acid material and positively charged inorganic nanoparticle, direct conjugation of nucleic acid onto the inorganic particle with a stimuli-responsive linker, and addition of cationic amphiphilic polymer to facilitate the assembly formation between the inorganic nanoparticle and the nucleic acid (Loh et al., 2015).

Another approach to protect and deliver nucleic acid cargos is via encapsulation using metal-organic frameworks (MOFs) (Liang et al., 2015; Li Y. et al., 2019; Poddar et al., 2019; Tolentino et al., 2020). These are porous structures built from metal ions or metal clusters linked by organic ligands (Li G. et al., 2019). The nucleic acid can be accommodated in the MOF structure through electrostatic and coordination interactions. Such physical confinement and the characteristic positive surface charge of MOFs offer effective protection of nucleic acid cargo against enzymatic degradation, which is, in many ways, analogous to viral capsids (Li Y. et al., 2019; Poddar et al., 2019).

While viruses deliver their nucleic acid cargo mostly through vesical fusion with the aid of some membrane fusion proteins (Harrison, 2008), inorganic nanoparticles do so with more complexity and hence present some formidable challenges. To achieve intracellular response, the nucleic acid cargo preferably needs to disassemble from the inorganic nanoparticle construct and escape the endosome. The mechanism by which these events (cell internalization and endosomal escape) occur depends on the identity and properties of the inorganic core, chemistry of the conjugation technique utilized, and response of other nanoparticle components to cellular or external stimuli (Sokolova and Epple, 2008). For example, magnetic iron oxide (Fe_3_O_4_) nanoparticle, when utilized as a delivery vehicle, can be stimulated to produce oscillating magnetic fields which could then promote more efficient endocytosis (Fouriki and Dobson, 2014). Furthermore, the inclusion of cell penetrating peptides and cationic amphiphilic polymers (e.g. polyethylenimine) as transfecting components assists in the endosomal escape via membrane destabilization and osmotic swelling, respectively (Thomas and Klibanov, 2003; Dowaidar et al., 2017). On the other hand, biocompatible MOFs like Zeolithic Imidazolate Framework-8 (ZIF-8) possess a hydrophobic and positively charged surface (Zhuang et al., 2014), which enable them to interact with the cell membrane and enable internalization through endocytosis.

A promising use of a metal nanoparticle for nucleic acid delivery is exemplified by spherical nucleic acids (SNAs). SNAs radially display a high density of nucleic acids around a spherical nanoparticle. The introduction of high concentrations of salt masks the polyanionic backbone of the nucleic acids, permitting clustering around a very small surface area (Mirkin et al., 1996; Cutler et al., 2011; Cutler et al., 2012). Moreover, the attachment of nucleic acids to a scaffold enhances their target binding affinity to complementary nucleic acids by restricting their conformational flexibility, reducing the entropic cost of binding (Lytton-Jean and Mirkin, 2005). SNAs have low immunogenicity (Massich et al., 2009) and are readily taken up by cells (Cutler et al., 2011) via caveolin-dependent endocytosis (Choi et al., 2013), eliminating the need for potentially toxic transfection agents (Cutler et al., 2011; Cutler et al., 2012). Unlike the abovementioned examples of inorganic nanoparticles, SNAs do not rely on complexation nor encapsulation to protect their nucleic acid cargo (Mirkin et al., 1996; Cutler et al., 2011; Cutler et al., 2012). The mechanism by which they protect nucleic acids is discussed more in Section *Self-Generated Sterics*.

#### Self-Generated Sterics

The overall 3D architecture of spherical nucleic acids (SNAs) imparts nuclease resistance through steric-shielding and enhanced local ionic strength (Seferos et al., 2009). This sterics-based mechanism of nucleic acid protection has defined an entire class of nucleic acid delivery systems. These nucleic acid displaying nanomaterials or NADNs, have recently been reviewed by Gudipati et al. (2019). While the metallic gold core provides a means of sensing and tracking the intracellular fate of the nanoconstructs (Mirkin et al., 1996; Cutler et al., 2012), it has limited therapeutic use. Thus, later generations of SNAs that have been developed contain biocompatible cores such as such proteins (Brodin et al., 2015; Samanta et al., 2020) and liposomes (Banga et al., 2014).

Designed to build upon the successful properties of SNAs, NADNs utilize densely packed oligonucleotides around a scaffold, enhancing oligonucleotide stability and permitting scavenger-mediated endocytosis but are built upon biodegradable core materials. The scaffolds of reported NADNs are chemically diverse (Rush et al., 2013; Banga et al., 2014, 2017; Awino et al., 2017; Ding et al., 2018; Roloff et al., 2018; Ruan et al., 2018) and can be programmed for responsiveness to biochemical stimuli (Awino et al., 2017; Santiana et al., 2017). For example, our lab developed nucleic acid nanocapsules (NANs) comprised of nucleic acids photochemically tethered to the surface of stimuli-responsive, crosslinked micelles (Awino et al., 2017; Santiana et al., 2017).

Overall, this section underscores that virus particles are metastable machines built to protect the viral genome and that its overall responsiveness to the environment enables it to carry out its function as an infectious particle. In a similar fashion, nonviral synthetic carriers are designed to protect nucleic acid cargo and facilitate controlled release. Table 1 provides a summary of the structures and cellular trafficking of viral and nonviral carriers. Similar to viruses, functional components (as summarized in Table 2) are incorporated into the design of nonviral vectors that facilitate cellular entry (Section *Cellular Targeting, Attachment, and Entry*), endosomal escape (Section *Cytosolic Delivery*), and nuclear delivery (Section *Nuclear Delivery*).

**TABLE 1:** Nucleic acid carriers: Properties and trafficking.

Vector	Core design	Mode of entry	Endosomal escape	Nuclear delivery	Nucleic acids delivered	Ref
**Viruses and virus-like particles**						
HIV	Enveloped, cone shaped capsid size: 100 nm	Sequential binding of spike protein GP120 to CD4 and a chemokine receptor promotes membrane fusion and direct cytosolic delivery.	N/A	Preinitiation complex is transported along the microtubule to the perinuclear region. NLS peptides on viral capsid promote karyopherin- mediated nuclear uptake.	DNA, siRNA, shRNA, miRNA	Bukrinsky (2004); Hamid, et al. (2015); Fanales-Belasio et al. (2010)
CCMV	Non-enveloped, icosahedral capsid size: 30 nm	Direct cytosolic delivery	N/A	N/A	siRNA, mRNA, dsDNA	Lam and Steinmetz (2019); Pretto and van Hest (2019); Villagrana-Escareño et al. (2019); Mukherjee et al. (2006)
MS2	Non-enveloped bacteriophage with complex structure and icosahedral head size: 27 nm	Receptor-mediated endocytosis (when targeting ligands are added)	Incorporation of penetrating or fusogenic peptides could facilitate endosomal escape.	N/A	shRNA, mRNA, miRNA, siRNA	Fu and Li (2016); Galaway and Stockley (2013); Ashley et al. (2011); Prel et al. (2015); Yao et al. (2015); Pan, et al. (2012a); Pan et al. (2012b); Lam and Steinmetz (2018)
M13	Non-enveloped filamentous bacteriophage composed of helically arranged coat proteins size: 880 nm length, 6.6 nm width	Receptor-mediated endocytosis (when targeting ligands are added)	Disruption of caveosomes and/or caveosome trafficking (need further studies)	N/A	Mammalian DNA transgene	Kim et al. (2012); Tian et al. (2015); Karimi et al. (201); Moon et al. (2015); Passaretti et al. (2020); Yata et al. (2014)
AAV	Nonenveloped, icosahedral capsid size: 20–25 nm	Clathrin-mediated endocytosis	Endosomal acidification exposes phospholipase domain that lyses endo-lysosomal membrane	Endosomal acidification exposes NLS domains that direct genes to nucleus	siRNA, DNA	Tomar et al. (2003); Xu et al. (2005)
AdV	Nonenveloped, icosahedral capsid with fiber knobs on vertices size: 90–100 nm	Binding to CAR and integrins facilitates integrin-dependent endocytosis	unknownCeramide-enhanced insertion to and membrane disruption of early endosomes by protein VI	Microtubule dynein/ dynactin motor complex	DNA transgene, therapeutic genes	Greber et al. (1997); Tatsis and Ertl (2004); Volpers and Kochanek (2004); Russell (2009); Fay and Panté (2015); Staring et al. (2018)
IV	Enveloped, spherical capsid with helical symmetry size: 80–120 nm shape: Spherical	Binding to sialic acid groups facilitates endocytosis.	pH drop in endosomes reveals hydrophobic HA2 subunit that mediates fusion	NLS sequences on nucleoprotein mediate karyopherin -dependent nuclear delivery	siRNA, miRNA	James and Whitley (2017); Couch (1996); Mammen et al. (1998); Pinto, et al. (1992); Neumann et al. (1997); Li et al. (2015); de Jonge et al. (2006); Li H. et al. (2013)
HBV	Enveloped, icosahedral capsid size: 42 nm	Binding of major surface antigens of HBV to cellular receptors NTCP and HSPG facilitate receptor mediated endocytosis.	Need further studies but shown to be insensitive to pH	Microtubule assisted perinuclear delivery; karyopherin-dependent nuclear entry	DNA	Li (2015); Venkatakrishnan and Zlotnick (2016); Tsukuda and Watashi (2020); Brandenburg et al. (2005)
EBOV	Enveloped, filamentous virus with helical symmetry Diameter: 80 nm, length: 600–1,400 nm	Macropinocytosis	Binding to NPC1 in late endosomes or lysosomes facilitates fusion and endosomal escape	N/A	none	Beniac et al. (2012); Falasca et al. (2015); Hunt, et al. (2012); Kondratowicz et al. (2011); Nanbo et al. (2010); Aleksandrowicz et al. (2011); Carette et al., 2011; Côté et al., 2011; Wang et al. (2016a)
SV40	Non-enveloped, icosahedral capsid size: 45 nm	SV40 VP1 protein binds to MHC-1 receptor and undergoes caveolin mediated internalization	Caveosomes undergo dynamic shape changes, and the virus is transported to the smooth endoplasmic reticulum.	Capsid disassembly occurs in smooth ER; exposed NLS peptide facilitates nuclear uptake via karyopherin -mediated pathway	none	Fay and Panté 2015, Norkin et al. (2002), Anderson et al. (1998), Martini et al. (2007), Pelkmans et al., 2001, Nakanishi et al. (2007)
**Carbohydrate-based vector**						
siRNA-GalNAc3 conjugates	Tris-GalNAc ligand of ASPGR is covalently attached to siRNA	Receptor-mediated endocytosis	Unknown	N/A	siRNA	Nair et al. (2014); Springer and Dowdy (2018)
**Protein/Peptide-based vectors**						
ARCs	Antibody is conjugated to alkyne-siRNA sense strand via a bifunctional azidoLys peptide linker	Receptor-mediated endocytosis	N/A	N/A	siRNA	Huggins et al. (2019)
REDV-Gm-TAT-Gm-NLS tandem peptide	Peptide sequences covalently linked with Gly repeats pack pDNA via electrostatic condensation size: 200 nm shape: Spherical	REDV selectively binds to integrin α4β1 of endothelial cells, leading to endocytosis. TAT promotes membrane permeability.	NLS have buffering capacity	NLS facilitates karyopherin α/β mediated perinuclear delivery	pDNA	Hao et al. (2017)
T-Rp3	Modular His6-tagged protein composed of the recombinant DBP, a DBD, and TAT size: 100 nm shape: free from-toroidal; bound form-spherical	TAT facilitates endocytosis mostly via clathrin-dependent pathway	His6 tag induces “proton-sponge effect”	T-Rp3 interacts with microtubule and is transported to the perinuclear region nuclear entry is due to hydrophobic interaction of positively charged amino acid residues with NPC	pDNA, siRNA, dsRNA	Favaro et al. (2014); Favaro et al. (2018)
**Polymer-based vectors**						
A-C3	Cationic diblock copolymer pDMAEA-PImPAA-pBA condenses nucleic acids size: 200 nm shape: Spherical	Cationic pDMAEA facilitatesclathrin-mediated endocytosis	Ionizable PImPAA elicits proton sponge effect; hydrophobic PBA inserts into endosomal membrane	BA binds to NPC via hydrophobic interaction	pDNA, siRNA	Gillard et al. (2014), Truong et al. (2013)
PAT-SPN	Cationic diblock copolymer DMAEA-PAA-BA condenses nucleic acids; PEG shell is tethered to polyplex core through an MMP-7 peptide substrate size: 46 nm shape: Spherical	MMP-7 activated particle enter via endocytosis	pH-dependent membrane destabilization by endosomolytic PAA-BAA block	Not shown	DNA, siRNA	Li H. et al. (2013)
**Lipid-based vectors**						
Liposomes	Lipid combinations containing ionizable cationic lipids, fusogenic lipids, cholesterol, and PEG-lipids form spherical bilayers with an aqueous core size: <200 nm shape: Spherical	Direct fusion or endocytosis	Membrane fusion – can be made responsive to cellular (pH, enzymes, redox potential) or external (temperature, magnetic field, light) stimuli; may also be decorated with penetrating or fusogenic domains to facilitate escape	N/A	mRNA, siRNA, pDNA, ASOs	Semple et al. (2010); Akinc et al. (2010); Corbett et al. (2020); Callaway (2020); Jeffs et al. (2005); Wheeler et al. (1999); Lechardeur et al. (1999); Heidarli et al. (2017)
SLNPs	Nucleic acids combined with cationic lipids form neutral complexes that are encapsulated by solid lipids size: ∼150 nm shape: Spherical	Phagocytosis or endocytosis (depends on cell type and surface modification)	Membrane destabilization	N/A	siRNA	Lobovkina et al. (2011); Arana et al. (2019)
**Inorganic nanoparticles**						
AuNPs	Covalent attachment of nucleic acid cargo or supramolecular assembly size: ∼50 nm shape: Spherical, rod-like, star-like, triangular	Clathrin-mediated endocytosis	Polycationic functionalities on the surface disturb the pH balance leading to osmotic swelling and endosomal rupture - “proton sponge” mechanism	N/A	DNA, siRNA, miRNA	Burger et al. (2014); Ding et al. (2014a); Neshatian et al. (2014); Mendes et al. (2017); Xie et al. (2017)
Fe_3_O_4_ NPs	Covalent attachment of nucleic acid cargo or supramolecular assembly size: 50–100 nm shape: Spherical	Endocytosis that could be enhanced by the application of oscillating magnetic field	Osmotic swelling if polycationic polymers are used, membrane destabilization if coated with lipids or functionalized with cell penetrating peptides	N/A	DNA, siRNA	McBain et al. (2008); Cutler et al. (2010); Jiang et al. (2013); Urie and Rege (2015); Dowaidar et al. (2017); Cruz-Acuña et al. (2018)
NanoMOFs	Biomineralization, pore encapsulation,supramolecular assembly size: 30–300 nm shape: Spherical, ellipsoidal, cubic, hexagonal, octahedral	Endocytosis	Osmotic swelling induced by metal cations from degraded MOF	N/A	DNA, aptamers (DNA and RNA), miRNA, siRNA, pDNA	Liang et al. (2015); Peng et al. (2018); Sun et al. (2018); Li Y. et al. (2019); Teplensky et al. (2019); Sun et al. (2020)
NPSCs	Complexes of nucleic acid and Arg-rich inorganic nanoparticles are assembled on an oil drop size: 150–500 nm shape: Spherical	Direct fusion and cytosolic delivery	N/A	No data yet	siRNA, CRISPR-Cas9-gRNA	Jiang et al. (2015); Mout et al. (2017); Jiang et al. (2018)
usAuNP	Tiopronin-covered AuNPs conjugated to TFO size: 2–20 nm shape: Spherical	Caveolae-mediated endocytosis	Passive diffusion out of the endosome	2 and 6 nm gene carrying NP undergo passive diffusion whereas any size above 10 nm stays in cytoplasm.	c-myc promoter-binding TFO	Cai et al., 2011; Huang et al. (2012); Huo et al. (2014)
**Nucleic acid displaying nanostructures (NADNs)**						
SNAs	Outward display of densely packed nucleic acids physically adsorbed or covalently bonded to a nanoparticle core size: <100 nm shape: Spherical, rod-like, triangular prism	Caveolae-mediated endocytosis	N/A, most trapped in endosomes	N/A	siRNA, miRNA, DNAzymes, aptamers, ribozymes, immunostimulatory DNA	Mirkin et al. (1996); Elghanian et al. (1997); Jin et al. (2003); Rosi et al. (2006); Massich et al. (2009); Seferos et al. (2009); Cutler et al. (2011); Cutler et al. (2012); Young et al. (2012); Choi et al. (2013); Banga et al. (2014); Banga et al. (2017); Li et al. (2018); Rouge et al. (2015)
NANs	Nucleic acids are radially displayed on and photochemically tethered to the surface of crosslinked micelles. Hollow core permits co-delivery of small molecules and large biomolecules size: 20–180 nm shape: Spherical	Endocytosis	Micelle cross-linkages are enzymatically cleaved by endosomal esterases or proteases, revealing a hydrophobic surfactant tail that facilitates cytosolic access	N/A	DNA, siRNA, DNAzyme, pDNA	Awino et al. (2017); Santiana et al. (2017); Hartmann et al. (2018); Hartmann et al. (2020); Tolentino et al. (2020)
Nucleic acid Nanogel	Double stranded nucleic acid linkers with single stranded overhangs hybridize with multiple DNA strands clicked onto a polymeric backbone, serving as crosslinks that condense the construct into a nanogel size: 80–1,200 nm shape: Spherical	Endocytosis	Unknown	None	siRNA, Cas9/sgRNA	Ding et al. (2018); Ding et al. (2019); Ding et al. (2020)

Abbreviations: AAV, adeno-associated virus; siRNA, small interfering RNA; AdV, adenovirus; shRNA, small hairpin RNA; VLP, virus-like particle; NTPC, sodium taurocholate cotransporting polypeptide; HSPG, heparan sulfate glycoprotein; CCMV, cowpea chlorotic mottle virus; mRNA, messenger RNA; miRNA, microRNA; GalNAc, N-acetylgalactosamine; ASPGR, asioglycoprotein receptor; ARC, antibody-RNA conjugate; REDV, Arg-Glu-Asp-Val; G_m_, Gly repeats; TAT, transactivator of transcription peptide; NLS, nuclear localization sequence; pDNA, plasmid DNA; DBD, DNA-binding domain; DBP, dynein-binding protein; pDMAEA, dimethylaminoethyl methacrylate; PImPAA, P(N-(3-(1H-imidazol-1-yl)propyl)acrylamide; pBA, poly (butyl acrylate); PAT-SPN, proximity-activated targeting smart polymeric nanoparticle; PEG, polyethylene glycol; MMP-7, matrix metalloproteinase-7; SLNP, solid lipid nanoparticle; AuNP, gold nanoparticles; Fe_3_O_4_ NP, iron oxide nanoparticle; NanoMOF, nano metal-organic framework; NPSC, nanoparticle stabilized nanocapsules; CRISPR-Cas9-gRNA, clustered regularly spaced palindromic sequences (CRISPR) CRISPR-associated (Cas9) guide RNA; usAuNP, ultrasmall gold nanoparticle; TFO, triplex forming oligonucleotides; SNA, spherical nucleic acids; NAN, nucleic acid nanocapsules.

**TABLE 2:** Key components added to modulate trafficking.

Components	Examples	Mechanism of action	Nucleic acid carriers	Ref
**Targeting, attachment, and entry**				
Aptamers	Electrostatically adsorbed RNA-based CD30 aptamer	Binding to surface CD30 specifically overexpressed in ALK + ACLC promotes endocytosis	siRNA-loaded cationic polymer-based vector	Zhao et al. (2011)
	Surface-anchored RNA-based transferrin aptamer	Binding to cell surface transferrin receptor mediates endocytosis	siRNA-loaded liposomes	Wilner et al. (2012)
Peptides	Integrin-targeting peptides (e.g. RGD, REDV, AG86)	Binding to integrins facilitates clathrin- or receptor- mediated endocytosis	siRNA-peptide conjugates, pDNA-peptide complexes. siRNA-loaded liposomes	Yonenaga et al. (2012); Hao et al. (2017); Kang et al. (2019)
	GLP1	Binding to GLP1R on pancreatic islet beta cells facilitates endocytosis	ASO-GLP1 peptide conjugates	Ämmälä et al. (2018)
	TAT	Cationic naked or conjugated peptide can enter cells via macropinocytosis or receptor-mediated endocytosis	siRNA-TAT-EED conjugates	Lönn et al. (2016); Khan et al. (2020)
	R8	Acid-labile hydrazone linkages are cleaved around tumor cells, revealing cationic CPP that mediates endocytosis	siRNA-loaded, ACPP-decorated liposomes	Xiang et al. (2017)
	MPG	Hydrophobic domain of peptide facilitates direct cytosolic entry	Noncovalent MPG complexes peptide-siRNA and peptide-pDNA complexes	Simeoni (2003)
Carbohydrates	GalNAc	Multivalent binding to hepatocyte ASGPR mediates endocytosis	siRNA-GalNac conjugates	Nair et al. (2014)
Small molecules	Folate	Binding to folate-receptors overexpressed in cancer cells mediates endocytosis	pDNA loaded liposomes functionalized with folic acid as targeting ligand, miRNA-folate conjugates.	Sikorski et al. (2015); Cui et al. (2016); Orellana et al. (2017)
	Bivalent β-turn analogues	Mimic β-turn recognition motifs that facilitate protein-protein interactions; hydrophobic tail added to enhance membrane attachment	pDNA-loaded BIVs	Burgess (2001); Shi et al. (2010)
Antibodies	Surface-anchored Anti-CD3 and Anti-CD8 antibodies	Binding to surface CD3 and CD8 receptors on T-cells promotes endocytosis	mRNA-loaded polymer-based carrier	Moffett et al. (2017)
	Anti-CD22 mAb-SA	Binding to CD22 receptor in lymphoma cells promotes receptor-mediated endocytosis	siRNA-loaded polymer-based system	Palanca-Wessels et al. (2011)
	Surface-conjugated Anti-HER2 mAb	Binding to HER2 overexpressed in breast cancer cells facilitates endocytosis	siRNA-loaded inorganic- and polymer-based system	Ngamcherdtrakul et al. (2015)
	Anti-CD33 IgG4 mAb	Binding to CD33 ^+^ AML THP1 cells facilitates endocytosis	Antibody-siRNA conjugates (ARCs)	Huggins et al. (2019)
**Endosomal escape**				
Peptides	Fusogenic peptides (e.g. HA2-derived peptides, GALA, KALA)	Glu- or His-rich peptides undergo acid-driven conformational change to alpha-helical structure, leading to pore formation	pDNA entrapped in gelatin-silica nanoparticles modified with fusogenic peptides, or nanobiomimetic carrier composed of targeting and fusogenic peptides by which DNA is condensed.	Ye et al. (2012); Kusumoto et al. (2014); Alipour et al. (2017); Ni et al. (2019)
	Addition of 5–20 His to the targeting ligand	Proton sponge effect	pDNA-His modified peptide complexes	Lo and Wang (2008); Chang et al. (2010)
	Endosomal escape domains (EEDs)	Hydrophobic W- and F-containing peptides destabilize endo-lysosomal membranes	siRNA-TAT-EED conjugates	Lönn et al. (2016)
Small molecules	Oligonucleotide enhancing compounds (OECs)	Enhance membrane permeability	ASO/SSO/siRNA-OEC conjugates	Yang et al. (2015); Wang et al. (2017); Juliano et al. (2018); Seth et al. (2019)
	Cationic Amphilic drugs (CADs, e.g. chloroquine)	Weak bases that destabilize the endo-lysosomal membrane	Adjuvants for GalNAc-cholesterol-siRNA conjugates	Du Rietz et al. (2020)
	Nigericin	Ion exchange between endosomal H+ and cytosolic K+ results in endosomal swelling and rupture	miRNA-folate-nigericin conjugates	Orellana et al. (2019)
Polymer	PEI	Osmotic endosomal rupture	siRNA-loaded cationic polymer	Zhao et al. (2011)
	Multiblock (co)polymers (e.g. DMAEA-PAA-PBA, pDMAEA-PImPAA-PBA)	Endosomal rupture via ionic and hydrophobic interactions with membrane	DNA/RNA-polymer complexes	Li H. et al. (2013); Truong et al. (2013); Gillard et al. (2014)
Hydrophobic domains	Surfactant	Surfactant destabilizes endosomal membrane	Polymeric micelle, siRNA-DNA conjugates, DNAzyme-NANs	Zhang et al. (2015); Hartmann et al. (2018); Hartmann et al. (2020)
	Cationic or ionizable lipids (e.g. DOPE)	Lipid fusion destabilizes membrane	siRNA-loaded liposomes	Semple et al., 2010; Wilner et al. (2012)
**Nuclear targeting and entry**				
Aptamers	DTS (from SV40 enhancer region)	DTS binds to cytoplasmic NLS-tagged proteins bound for nuclear delivery	DTS sequence-containing plasmids	Miller and Dean (2009)
	NFκB-motif embedded on plasmid sequence	NFκB binds with motif on pDNA and shuttles construct to nucleus	pDNA/polymer complexes	Breuzard et al. (2008)
	Surface-displayed DNA-based nucleolin aptamer (AS411)	Active transport and binding to nucleolin localized in nuclear membrane	Polymeric micelle	Zhang et al. (2015)
Peptides	Dynein binding protein (DBP)	DBP binds to motor and is carried to centrosome through microtubules	Recombinant DBP-containing protein condensed with pDNA, siRNA and dsRNA	Favaro et al. (2018); Favaro et al. (2014); Dalmau-Mena et al. (2018)
	Nuclear localization signal (NLS)	Form weak, multiple interactions with cytoplasmic karyopherin bound for active nuclear transport via NPC	pDNA condensed with cationic NLS; AuNP conjugated complex of CRISPR/Cas9-gRNA, Cas9, and NLS; pDNA-NLS conjugates	Hao et al. (2017); Kim et al. (2017); Mout et al. (2017)
Small molecules	Dexamethasone (Dex)	Dex binds to nuclear membrane glucocorticoid receptor and dilates NPC; enhances affinity of polycations to nuclear membrane	HA/PEI1800-Dex/pDNA ternary complexes	Fan et al. (2013)

Abbreviations: CD, cluster of differentiation (receptor); ALK^+^, anaplastic lymphoma kinase; ACLC, anaplastic large cell lymphoma; siRNA, small interfering RNA; ASO, antisense oligonucleotide; GLP1, glucagon-like peptide 1; GLP1R, glucagon-like peptide 1 receptor; TAT, transactivator of transcription (peptide); EED, endosomal escape domain; CPP, cell-penetrating peptide; R8, Octa-Arg (peptide); GalNAc, N-acetylgalactosamine; ASGPR, asioglycoprotein receptor; BIV, bilamellar invaginated vesicle; miRNA, microRNA; mAb-SA, streptavidin-conjugated monoclonal antibody; HER2, human epidermal growth factor 2; IgG4, immunoglobin G4; AML, acute myeloid leukemia; HA2, hemagglutinin 2 (peptide); GALA, Glu-Ala-Leu-Ala (peptide); pDNA, plasmid DNA; SSO, splice-switching oligonucleotide; PEI, polyethylenimine; pDMAEA, dimethylaminoethyl methacrylate; PImPAA, P(N-(3-(1H-imidazol-1-yl)propyl)acrylamide; pBA, poly (butyl acrylate); PAA, propylacrylic acid; DOPE, dioleoylphosphatidylethanolamine; DTS, DNA nuclear targeting sequence; SV40, simian 40 virus; NFκB, nuclear factor kappa-light-chain-enhancer of activated B cells; dsRNA, double-stranded RNA; AuNP, gold nanoparticle; CRISPR-Cas9-gRNA, clustered regularly spaced palindromic sequences (CRISPR) CRISPR-associated (Cas9) guide RNA; NPC, nuclear pore complex; HA, hyaluronic acid.

## Cellular Targeting, Attachment, and Entry

Tropism is the ability of viruses to target specific cell types by binding their surface protein or peptide ligands to specific host cell receptors. The elaborate means with which they make use of these ligands accounts for their cell target specificity and high uptake efficiency (Ni et al., 2016). Mechanisms governing the targeting and specific uptake of viruses and nonviral vectors alike rely on the use of electrostatic forces, multiple receptors for enhanced specificity, and multivalent interactions.

### Receptor Ligands are Central to the Molecular Mechanisms of Targeting, Attachment, and Entry

Prior to entry, viruses often adhere to the cell surface via non-specific electrostatic interactions involving viral surface components (i.e. membrane glycoproteins) and negatively charged sugars (i.e. heparin sulfate) attached on the target cell surface (Grove and Marsh 2011; Mazzon and Marsh 2019). Though such interactions may lack specificity, they provide the virus an initial foothold on the cell before recruiting specific cell receptors and facilitating entry (Grove and Marsh 2011). Most viruses, which include influenza virus, coronavirus, reovirus and polyomavirus, utilize the sialic acid receptors on the host cell surface for initial attachment (Maginnis 2018). Taking inspiration from this virus behavior, a number of delivery methods have either functionalized nucleic acid cargo with sialic acid (St-Pierre et al., 2016) or encapsulated them in nanocarriers decorated with sialic acids on the surface (Tang et al., 2019). A notable example of the latter strategy is demonstrated in the work of Tang et al. (2019). In their study, they have successfully delivered reporter (luciferase) and functional (antitumor p53) mRNAs to cancer cells using a liposomal nanoparticle containing surface sialic acids. Other than sialic acids, viruses utilize a plethora of receptor ligands which are proteoglycans (i.e. cell adhesion molecules) and lipids (i.e. PS) by nature, to mediate cellular attachment and entry (Maginnis, 2018). On the other hand, synthetic vectors make use of a more chemically diverse array of ligands but mostly for targeting purposes.

Targeted delivery is desired for synthetic vectors as it confers safety, efficacy, and efficiency. It limits the release of the therapeutic to diseased cells or tissues, minimizing adverse off-target effects that could outweigh therapeutic benefits. Secondly, it enhances efficacy by localizing a high concentration of the drug to a specific site. Third, efficiency is achieved by providing access to sites such as certain cells or subcellular locations (e.g. nucleus) that are normally inaccessible to the therapeutic (Rohovie et al., 2017). Many non-viral strategies have derived targeting domains from viral ligands for specific cell or tissue targeting. For example, the adenovirus-derived RGD peptide has been used to direct the nucleic acid delivery of lipoplexes, dendriplexes, and polyplexes to tumor cells overexpressing integrin α_v_β_3_ on the cell surface (Danhier et al., 2012). The successful delivery of RGD-conjugated ASOs to melanoma cells has also been demonstrated (Alam et al., 2008; Juliano et al., 2008; Kang et al., 2008; Juliano et al., 2011). An RGD-based polycationic liposome was also developed to specifically target cancer cells and angiogenic endothelial cells (Yonenaga et al., 2012).

Other ligands of non-viral origin also offer targeting properties. For example, monoclonal antibodies have a been highly effective at targeting delivery of cytotoxic drugs to cancer cells (Sievers et al., 2001; Krop et al., 2010; Younes et al., 2010). Their ability to specifically and avidly bind to cell-specific receptors makes them equally viable targeting domains for biologics such as therapeutic nucleic acids. Their use in directing nucleic acid carriers has been demonstrated in several studies (Palanca-Wessels et al., 2011; Ngamcherdtrakul et al., 2015; Moffett et al., 2017; Huggins et al., 2019; Nanna et al., 2020). They can be either directly conjugated to the nucleic acid (Huggins et al., 2019; Nanna et al., 2020) or to the vector (Palanca-Wessels et al., 2011; Ngamcherdtrakul et al., 2015; Moffett et al., 2017). Antibody-RNA conjugates (ARCs) are promising in that they overcome possible limitations of nanoparticle-based formulations such as poor diffusivity, toxicity, and immunogenicity while still significantly extending the half-life of the cargo (Nanna et al., 2020). Earlier conjugation methods for therapeutic attachment to antibodies involve nonselective conjugation to lysine or cysteine residues. Consequently, prior formulations suffer mainly from product heterogeneity (Huggins et al., 2019). Recently published works on ARC synthesis involved highly specific mechanisms for conjugation, giving a precise drug:antibody ratio of 2 (Huggins et al., 2019; Nanna et al., 2020).

Nucleic acid aptamers offer another promising approach in delivering nucleic acid cargos to specific cell-types (Dassie and Giangrande, 2013). Aptamers are short, chemically synthesized, single stranded oligonucleotides (DNA or RNA), which adopt a specific three-dimensional (3D) structure and bind to their ligands with high affinity (K_D_s in the pico-to nano-molar range) (Sun et al., 2014). Although aptamer-nucleic acid conjugates possess no innate mechanisms for endosomal escape on their own, aptamers can be conjugated on to nucleic acid carriers with endosomal escape activity as a way to improve cell specific targeting (Yan and Levy, 2018). For example, Zhao et al. (2011) designed a nanocomplex composed of a cationic PEI core endosomal escape component, CD30 RNA aptamer targeting lymphoma cells and siRNA that inhibits the expression of anaplastic lymphoma kinase (ALK). Such an assembly was proven to selectively bind lymphoma cells, deliver the siRNA intracellularly, silence ALK expression, and arrest the growth of lymphoma cells (Zhao et al., 2011).

Lastly, small molecules are commonly used as targeting ligands as they are easily synthesized at a modest cost. They are more stable than biological ligands such as aptamers and peptides, and their conjugation is often relatively simple. However, these molecules are often not the natural ligands of the target cell receptors and thus have lower affinity and specificity for a given receptor, the latter giving rise to off-target effects. Nevertheless, the relative structural simplicity and functional designability of small molecules make them attractive and viable targeting domains (Friedman et al., 2013).

For example, folate (Vitamin B9) is widely used for targeting folate receptor-positive cell lines, with a high affinity (K_D_ = 1 nM) and minimal toxicity. Folate-functionalized vectors are typically internalized via receptor-mediated endocytosis, but reduced folate carriers, though having lower affinity, directly enter the cytosol. Folate-expressing imaging agents are currently in Phase I and Phase II clinical trials, but they are not yet clinically approved for targeting therapeutic nanoparticles (Sikorski et al., 2015).

Likewise, benzamides (anisamide, in particular) target sigma receptors that are upregulated in cancer cell lines. Benzamide analogues can also target dopamine receptors selectively. So far, these have been used to deliver small molecule drugs such as doxorubicin encapsulated in liposomes but have not been explored in gene-delivery yet (Banerjee et al., 2004; Mach et al., 2004).

### Multivalent Interactions Facilitate Cellular Uptake

Multivalent interactions between the viral ligands and host cell surface receptors not only amplify the strength of the interaction but also promote viral entry. This is exemplified by the influenza virus where the interaction of multiple capsid protein trimers (2-4 per 100 nm^2^) with spatially concentrated sialic acid functionalities on the surface of the host cell (50–200 per 100 nm^2^) is necessary for effective attachment and uptake (Mammen et al., 1998). Apart from high surface density, the spatial arrangement of the ligands is equally important. For example, the internalization of the simian virus 40 (SV40) necessitates the pentameric presentation of its viral capsid protein one to successfully bind to the cell-surface GM1 receptors and facilitate endocytosis (Ewers et al., 2010).

This parallels with carbohydrate-based delivery systems such as siRNAs and ASOs conjugated to N-acetylgalactosamine (GalNAc) for hepatic targeting. GalNAc involves multi-site interactions with asioglycoprotein receptors (ASPGR) of hepatocytes, facilitating endocytosis. (Nair et al., 2014; Debacker et al., 2020). In 2019, Alnylam’s givosiran (GIVLAARI^®^) was the first US Food and Drug Administration approved GalNAc conjugate for acute hepatic porphyria, and other conjugates are underway (Debacker et al., 2020). ASPGR is a liver-specific receptor that has been targeted for hepatic-directed therapeutics. It is a heterooligomeric complex that is capable of interacting with multiple GalNAc molecules (Meier et al., 2000). The strong binding affinity of monomeric GalNAc with ASPGR is in the micromolar range, and the avidity of the interaction can be enhanced by 10^3^ to 10^5^, depending on the number and spacing of GalNAc units (Lee and Lee 2000). Specifically, the structure of ASPGR was found to optimally bind three divergent GalNAc residues (Lee and Lee 2000) spaced from a common branch point by 14–20 Å and separated from each other by 15–20 Å (Lee et al., 1983; Khorev et al., 2008).

Other synthetic vectors having multivalent interactions with cell receptors have been developed to mimic viral behavior and have shown an enhanced cellular uptake of the carriers or nucleic cargo. A prime example of this is the study of Nakagawa et al. (2010), wherein they delivered a splice switching antisense oligonucleotide (SSO) directly conjugated to anisamide, a sigma receptor present in plasma membranes, to tumor cells, and investigated their ability to modify the splicing of a reporter gene (luciferase). Mono-anisamide and tri-anisamide conjugates were synthesized, and it was demonstrated that the multivalent conjugate yielded a more enhanced receptor-specific cell uptake and biological effect (Nakagawa et al., 2010). Another study highlighting the beneficial effect of multivalency to nucleic acid cargo internalization is carried out by Kang et al. (2018). In their study, siRNA specific to Bcl2, an anti-apoptotic protein, was tethered to MUC-1- and nucleolin-targeting aptamers and delivered to cancer cells. Fluorescence microscopy revealed the positive correlation between aptamer valency (n = 1, 3, 9) and cellular internalization. Moreover, higher tumor accumulation was observed for multivalent aptamer conjugates compared to mono- and divalent conjugates. These studies underscore the critical need for multivalent interactions in designing delivery systems for nucleic acids.

### Attachment to Multiple Receptors Confers Cell Target Specificity and Uptake Efficiency

Maginnis (2018) provides a comprehensive review of how virus interactions with host receptors govern pathogenicity. Worth noting are evolutionarily conserved mechanisms among viruses, redundancy in target primary receptors, and diversity of secondary receptors. One conserved mechanism is the conformational change involved in the sequential binding to multiple receptors that leads to fusion or endocytosis. For instance, the trimeric glycoprotein (GP) complex of the human immunodeficiency virus (HIV) is formed by the GP120/GP41 heterodimer and is necessary for cellular targeting and entry. GP120 binds CD4 on the surface of T-cells, T-cell precursors, macrophages, dendritic cells, and microglial cells. GP120 binding induces a conformational shift in the trimeric GP, revealing a GP120 binding domain specific for one of many chemokine coreceptors such as CXCR4 and CCR5. These coreceptors vary across different cells and thus mainly determine tropism (Fanales-Belasio et al., 2010; Wilen et al., 2012). The involvement of coreceptors form the basis of some anti-viral drugs such as Maraviroc, a US Food and Drug Administration and European Medicines Agency approved HIV/AIDS treatment. It acts by antagonizing CCR5, the secondary receptor of HIV in CD4^+^ T cells. In particular, maraviroc binding induces a change to the inactive conformer of CCR5 (López-Huertas et al., 2017).

In terms of redundant receptors, integrins are of particular interest because they are commonly involved in the internalization of viruses. Integrins are heterodimeric cell surface receptors that mediate cell adhesion, migration, differentiation, and tumor growth. The binding of a virus to a host induces the clustering and/or structural changes of integrins, resulting in intracellular cues that enhance binding affinity, drive structural changes in the cytoskeleton, and/or facilitate uptake. This is demonstrated by certain viruses such as the adenovirus whose secondary attachment to integrins initiates intracellular signals that ultimately lead to viral uptake (Stewart and Nemerow, 2007). For the human cytomegalovirus, the binding of its glycoproteins to both the epidermal growth factor receptors (EGFR) and integrin on the host cell brings EGFR and integrins into close proximity, eliciting signaling responses that facilitate cellular uptake and nuclear trafficking (Wang et al., 2005).

**FIGURE 3:**
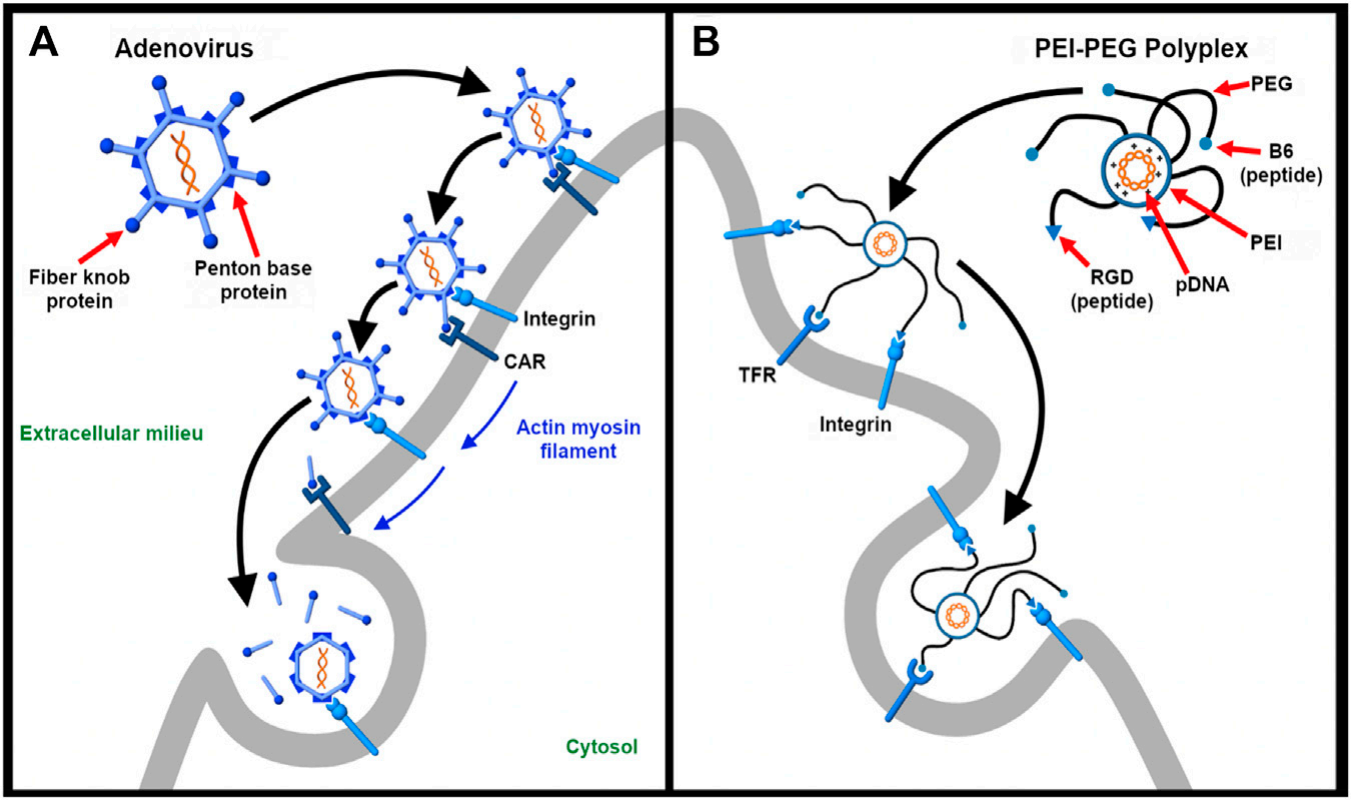
Targeting multiple receptors enhances cellular specificity and transfection efficiency. **(A)**. The entry of adenovirus into the host cell occurs in a three-step process – binding, drifting, and shedding. First, the adenovirus binds to the Coxsackievirus and adenovirus receptor (CAR) of the host cell surface through fiber knobs jutting out the vertices of the icosahedral shaped viral capsid. Second, acto-myosin drifting of the virus-bound CAR receptor leads to internment of the penton base protein of the viral capsid by integrins expressed on the cell surface. Third, the slow drifting motion (0.1 μm/s) of the CAR receptor and the stable nature of binding causes mechanical stress onto the viral capsid, the first uncoating step in the capsid disassembling process. The protein VI of the inner capsid is exposed which makes lesions in the plasma membrane and undergoes integrin-dependent endocytosis (Burckhardt et al., 2011) **(B)**. As described by Nie et al. (Nie et al., 2011), a synthetic dual-ligand targeted vector system was constructed using a cationic polymer PEI to deliver pDNA. PEG moieties were used to shield the charge of the polyplex. Inspired from natural viruses, the polyplex was conjugated with Transferrin receptor (TFR)-binding B6 peptide and integrin-recognizing RGD sequence for dual targeting purpose. The receptor specificity of the dual targeted polyplex shows increased gene transfection as compared to the single targeting peptide. The integrin receptor binding helps in cellular association and the vector is internalized via TFR-mediated endocytosis.

For synthetic vectors, engaging multiple receptors presents an opportunity for programming more specific and efficient nucleic acid delivery systems. The use of multiple ligands for enhanced specificity and uptake is guided by knowing which receptors are overexpressed in the tissue or region of interest. Just as integrins are often implicated in virus entry, they have become popular targets for drug and gene delivery for their natural abundance, efficient endocytosis, and differential expression on a number of tumor cells and angiogenic endothelial cells (Wang et al., 2010; Juliano et al., 2011). For instance, Nie et al. (2011) developed a synthetic dual-ligand targeted vector in which plasmid DNA is condensed by polyethylenimine (PEI). In this study, they conjugated PEG-ylated PEI-based polyplexes with peptides B6 and arginylglycylaspartic acid (RGD) that target transferrin and integrin, respectively. This strategy exploits the fact that tumor cells overexpress transferrin while vasculature that supply blood to these newly formed tumor cells overexpress integrins. Importantly, RGD-integrin binding stabilizes the B6-transferrin interaction. This design has shown to improve transfection efficiency and specificity. Thus, as illustrated in Figure 3, it demonstrates the power of mimicking the dual-receptor internalization of natural viruses such as the adenovirus, herpes simplex virus, and SV40 (Hussein et al., 2015).

In another study, Dong et al. (2018) depict the dual targeting ability of RGDK peptide sequence. In this particular example, they designed a siRNA/amphiphilic dendrimer complex decorated with a dual targeting peptide RGDK. The design of the targeting peptide is such that it protects and stabilizes the siRNA-dendrimer complex by electrostatic interaction. Similar to Nie et al.’s study, the RGD part binds to target integrin receptors on tumor vasculature while the full length RGDK interacts with neuropilin-1 (Nrp-1), which is expressed on tumor cells, thereby enhancing cellular uptake.

## Cytosolic Delivery

For a virus to deliver its genome to the cytosol or nucleus, it needs to penetrate either the cellular membrane or a subcellular membrane within the cytoplasm such as the endo-lysosomal membrane. This section talks about how viruses and synthetic carriers alike manage to bring their nucleic acid cargo into the host cell interior with mechanisms to overcome cellular barriers.

### Direct Cytosolic Delivery

Some enveloped viruses such as HIV are able to directly translocate their genome into the cytosol via cell membrane fusion. As mentioned in Section *Attachment to Multiple Receptors Confers Cell Target Specificity and Uptake Efficiency*, the binding of the HIV glycoprotein to its primary receptor drives structural changes within the glycoprotein, facilitating a subsequent interaction with a coreceptor that then mediates viral entry (Wilen et al., 2012). Binding to two receptors enhances the strength of viral attachment (Grove and Marsh, 2011; Ni et al., 2016), and for HIV, this allows the N-terminal fusogenic peptide of GP41 to penetrate the membrane. The heptad repeats of GP41 interact to form a hairpin loop, facilitating the fusion of the viral and host cellular membranes (Chan et al., 1997; Fanales-Belasio et al., 2010).

For nonviral carriers, a particle can also be designed such that it directly transfects cargo to the cytosol. For instance, Motion et al., (2012) reported a promising phosphatase-triggered liposome carrier that was directly inspired by HIV. It incorporates an inactive phosphorylated version of the GP41 peptide that, when dephosphorylated, shifts to its fusogenic alpha-helical conformer. The phosphorylated form, on the other hand, has an increased random coil structure that is unable to interact with a lipid membrane. Since phosphates are overexpressed and secreted by diseased tissues, the fusogenic peptide is activated in a diseased cell, facilitating fusion with the plasma membrane and targeted cytosolic delivery. Such system has great potential as a nucleic acid carrier. Additionally, studies have shown that exogenous miRNA (Vickers et al., 2011) and siRNA (Shahzad et al., 2011; Ding et al., 2014b) can be directly delivered to the cytosol of target cells using endogenous or reconstituted high density lipoprotein by targeting scavenger receptor B1 (Shahzad et al. 2011).

In addition, siRNA (Jiang et al., 2015; Jiang et al., 2018) and CRISPR-Cas9 ribonucleoprotein (CRISPR-Cas9-RNP) (Mout et al., 2017) can be directly transfected across the cell membrane using nanoparticle-stabilized nanocapsules (NPSCs). Previously shown to mediate the direct cytosolic delivery of small molecules (Yang et al., 2011) and proteins (Tang et al., 2013), NPSCs are formed by assembling a preformed complex of nucleic acids and arginine-coated nanoparticles on the surface of an oil droplet (Jiang et al., 2015). The inorganic- and lipid-based hybrid construct efficiently delivered nucleic acid cargo to the cytosol with an siRNA knockdown efficiency of 90% (Jiang et al., 2015; Jiang et al., 2018) and to the nucleus with a CRISPR-Cas9-RNP gene editing efficiency of 30% (Mout et al., 2017). *In vivo* assays of spleen-directed siRNA loaded NPSCs showed good selectivity and immunomodulatory activity, demonstrating the potential for targeted delivery (Jiang et al., 2018).

### Endosomal Escape

Most viruses and synthetic nucleic acid carriers are internalized via endocytosis. While viruses manage to escape into the cytosol efficiently, synthetic carriers pale in contrast (Ramamoorth and Narvekar, 2015), only having around 1–2% endosomal release (Gilleron et al., 2013). Thus, endosomal escape is the bottleneck of nucleic acid delivery and ultimately determines therapeutic efficiency (Gilleron et al., 2013; Shete et al., 2014; Selby et al., 2017).

While direct fusion with the plasma membrane may seem simpler, endocytosis offers several advantages—one being evasion of molecular crowding in the cytosol and microtubule-assisted shuttling to the nucleus or other subcellular locations (Barrow et al., 2013). Furthermore, as endocytosis is often linked to signaling cascades, the invading particle can influence its intracellular fate by targeting the appropriate receptor (Nemerow and Stewart, 1999; Marsh and Helenius, 2006). For viruses, endocytosis can lower the risk of triggering an immune response because rapid endocytotic uptake minimizes the exposure of viral immunogenic epitopes to the extracellular milieu (Miyauchi et al., 2009). Importantly, the physical integrity of the viral capsid is responsive to both chemical and mechanical stimuli brought about by interactions with the host. This provides a basis for disassembly once the genome has reached its target site (Greber 2016; Yamauchi and Greber, 2016). Similarly, endocytosis enables opportunities to embed responsiveness of a nonviral carrier to endolysosomal cues. For these reasons and the overwhelming tendency for nonviral carriers to undergo endocytotic entry, research efforts are more directed toward enhancing endosomal escape efficiency.

#### Cellular Cues Drive Endosomal Escape via Membrane Fusion or Penetration

Staring et al. (2018) provides an excellent discussion of how viruses carry out endosomal escape to avoid degradation or recycling. For their remarkable endosomal escape efficiency, viruses have served as templates for engineering the endosomal escape mechanism of non-viral vectors. A unifying theme is a conformational change in viral structural proteins that drives viral and endo-lysosomal membrane fusion for enveloped viruses or membrane penetration by nonenveloped viruses. These structural rearrangements are triggered by cellular cues such as low pH or acid-dependent proteolytic activity. Such viral proteins or peptides contain ionizable groups such as critical histidine residues whose imidazole groups (pKa∼6) are protonated as the pH drops in the endosome. These histidine residues act as pH sensors involved in pH-dependent structural changes of the protein or peptide as observed for the surface protein hemagglutinin (HA) glycoprotein (GP) of the influenza virus. Moreover, they also serve as internal buffers. This “proton sponge” effect leads to endosomal swelling and rupture. For this reason, histidine residues (5–20) are added to peptide domains (such as TAT) of nucleic acid carriers (Lo and Wang, 2008). A research study by Meng et al. (2016) has discussed a multifunctional peptide-based nanocarrier composed of different peptide fragments—a CPP segment (TAT) for cell penetration, an ELMD segment for endo-lysosomal membrane disruption, and stearyl moieties to improve hydrophobicity and cell membrane binding ability of the peptide-DNA complex. For the ELMD segment, six histidine resides were inserted to increase endosomal escape by “proton sponge” effect. All these amino acids were dextrorotatory to protect the DNA/peptide nanocarrier from proteolysis.

##### Membrane Fusion

**FIGURE 4:**
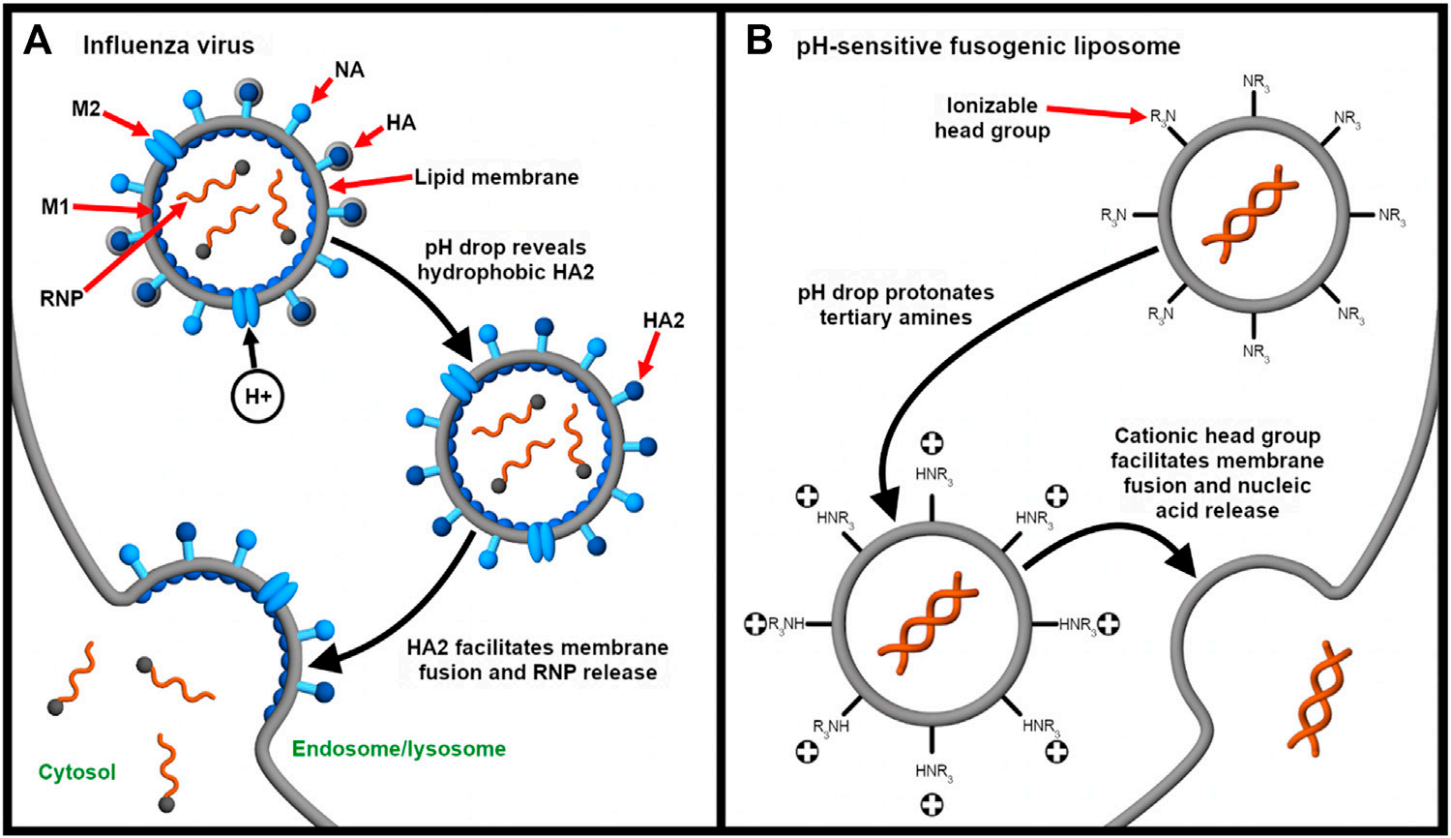
Endocytosis provides an opportunity for integrating stimulus-responsive nucleic acid release. **(A)** The influenza virus releases its genome (complexed with nucleoproteins, gray spheres) into the cytosol in a pH-dependent manner. Endosomal acidification drives the influx of protons through the Matrix Protein 2 (M2) ionophore. This liberates the ribonucleoprotein (RNP) complex from Matrix Protein 1 (M1) and exposes the fusogenic subunit HA2, which, in turn, facilitates fusion of the viral and endosomal membranes (Pinto et al., 1992). Neuraminidase (NA) enables release of the influenza virus from the host cell after replication (James and Whitley 2017). **(B)** On the other hand, pH-responsive fusogenic liposomes are composed of ionizable lipids with weakly basic head groups that are rapidly protonated as the pH drops in the endosomes. This enables the protonated lipids to promote fusion and nucleic acid release before lysosomal degradation (Budker et al., 1996; Kogure et al., 2008; Sato et al., 2012).

For the endosomal escape of enveloped viruses, the influenza virus is a classic model (Figure 4A). The fusogenic HA has been used or mimicked as an endosomal escape domain. Following endocytosis, the acid-triggered proteolysis induces the conformational change of the viral GP spike. This exposes the hydrophobic subunit HA2 that facilitates the endosomal escape of the ribonucleoprotein contents into the cytosol (Pinto et al., 1992). Specifically, endosomal acidification induces a conformational change in HA that sequesters charged residues glutamate-15 and aspartate-19. This reveals a V-shaped HA conformer with a hydrophobic pocket that penetrates deeply into the endosomal membrane. The enhanced penetration increases the lateral pressure in the hydrophobic pocket and the surface tension at the interface of the viral and endosomal membranes. Altogether, these drive the hemifusion of the two lipid membranes (Han et al., 2001).

Synthetic HA2 analogs have demonstrated improved endosomal escape ability (Ye et al., 2012). Ye et al. (2012) developed and studied different types of fusogenic peptides (HA2, R8, TAT, TAT-HA2, and TAT-R8) by conjugating them to gelatin-silica nanoparticles (GSNPs). These GSNPs were used to deliver plasmid DNA and their endosomal escape efficiency was measured and compared. They concluded that the endosomal escape efficiency of TAT-HA2 conjugate was superior as compared to others. Moreover, the concentration of the peptide dictates the extent of its interaction with the membrane. While the peptide domains only engage the membrane electrostatically at low concentrations, pore formation is observed at higher concentrations.

The endosomal escape of the influenza virus can be largely ascribed to the sequestering of the hydrophilic cap of HA to reveal a hydrophobic domain HA2 that then engages the endosomal membrane. This mechanism has inspired Lönn et al. (2016) to develop endosomal escape domains (EEDS), which are hydrophobic peptides containing tryptophan and phenylalanine residues. For EED-TAT-siRNA conjugates, the presence of indole and/or phenyl rings at an optimal distance of six PEG units from the TAT domain is able to significantly enhance the endosomal escape of siRNA. Additionally, the concept of hydrophobic unmasking has also been exhibited by nucleic acid nanocapsules. Amphiphilic surfactant-DNA conjugates were constructed to mimic the disassembly products of the nanocapsule. The membrane permeating ability of these conjugates (Hartmann et al., 2018) suggests that the hydrophobic group revealed only after disassembly could facilitate the endosomal escape of the degradation products.

Similarly, pH-sensitive fusogenic liposomes (Figure 4B) have been developed to mimic the acid-triggered endosomal escape of viruses (Budker et al., 1996). Sato et al. (2012) described the delivery of siRNA for gene silencing using low pH-activatable cationic liposomes. The responsiveness to low pH is enabled by using a lipid containing a tertiary amine head group that is almost neutral at physiological pH but is cationic at low endosomal pH (Moriguchi et al., 2005; Kogure et al., 2008; Sato et al., 2012). The lipid also consists of two long linoleyl fatty acid chains, forming cone-shaped molecules that further mediate endosomal escape through membrane fusion. Because the apparent pK of the ionizable lipid is 6.5, rapid membrane fusion and siRNA release is induced in the endosomes before lysosomal degradation occurs (Sato et al., 2012; Sakurai et al., 2014).

##### Membrane Penetration

Unlike enveloped viruses that possess a lipid envelope capable of fusing with the plasma or endo-lysosomal membrane, nonenveloped viruses make use of membranolytic peptides to escape the endosome. While membrane penetration is not completely understood, the exact mechanism can range from temporary membrane destabilization to pore formation to complete disruption (Staring et al., 2018). The elegance of viral endosomal escape using membranolytic peptides is exemplified by the adenovirus. The mechanical stress caused by binding multiple receptors primes the shedding of the capsid coat (Burckhardt et al., 2011). This liberates membranolytic viral protein VI that then creates small lesions on the plasma membrane. As a response, the host secretes lipid hydrolase acid sphingomyelinase that catalyzes ceramide production for membrane repair. The increased level of ceramide enhances interaction of protein VI with the endosomal membrane, leading to endosomal rupture. This illustrates how the host cell’s natural response to membrane damage is exploited by a virus for it to escape the limiting vesicle (Staring et al., 2018). Moreover, a study by Ortega-Esteban et al. (2015) showed that upon virus maturation, the expansion of the genome stiffens virions. As in the case of the adenovirus, the rise in internal pressure renders the capsid more susceptible to disruption and, thus, contributes to the overall endosomal escape mechanism and eventual uncoating of the virus at the nuclear pore complex (Ortega-Esteban et al., 2015; Greber 2016).

Similarly, the Glutamic acid-Alanine-Leucine-Alanine (GALA) peptide is a targeting and endosomal escape peptide that has been used in siRNA delivery (Subbarao et al., 1987; Kusumoto et al., 2013; Kusumoto et al., 2014). GALA was originally designed to undergo an acid-triggered change from a random coil to a membrane-disrupting alpha helical structure (Subbarao et al., 1987). Later on it was found to target the sialic acid residues on lung endothelium (Kusumoto et al., 2013), making it a promising multifunctional ligand. On the other hand, KALA is a modified version of GALA with alanine to lysine substitutions and reduced glutamic acid content. These features allow DNA condensation, endo-lysosomal disruption, and nucleic acid release (Wyman et al., 1997; Shaheen et al., 2011). Miura et al. (2017) performed a complete study of KALA as a fusogenic peptide. They modified the surface of a DNA-encapsulating liposome with KALA peptide sequences. In this study, they found that as compared to the full-length KALA sequence (27 residues), the short-KALA3 peptide (14 residues) was the shortest KALA peptide to form a α-helical structure at physiological pH. Thus, short-KALA3 can be used to elicit transgene expression (Miura et al., 2017). KALA peptide has also been used before for the delivery of siRNA-PEG conjugates (Mok and Park 2008).

#### Small Molecules for Enhancing Endosomal Escape Efficiency

The fact that fusogenic or membranolytic peptides are often required to gain cytosolic access underscores the necessity for an endosomal escape component in a drug delivery system. This idea has been extended to various small molecules that can be used as tools to cross the endo-lysosomal membrane either through direct conjugation to or co-delivery with the nucleic acid cargo (Gilleron et al., 2015; Osborn et al., 2015; Maxfield 1982; Juliano et al., 2018; Joris et al., 2018; Du Rietz et al., 2020; Yang et al., 2015; Wang et al., 2017). For example, cationic amphiphilic drugs (CADS) have been shown to enhance siRNA delivery due to their ability to increase the permeability of the endo-lysosomal membrane (Joris et al., 2018; Du Rietz et al., 2020). On the other hand, oligonucleotide enhancing compounds (OECs) are small molecules covalently linked to siRNAs, ASOs, and single stranded oligonucleotides and have been screened for improved cytosolic and nuclear delivery without an external carrier (Yang et al., 2015; Wang et al., 2017). Through a set of structure-activity experiments, hydrophobic phenyl rings, the presence and relative placement of a tertiary amine, and carbamate modifications were identified as essential and tunable features for enhancing the therapeutic availability of the oligonucleotides. How OECs influence the intracellular redistribution of oligonucleotides is not yet clear but, similar to CADs, involves an increase in endomembrane permeability rather than complete disruption. Though the potency imparted by OECs holds great promise, the challenge of enhancing efficacy while minimizing cytotoxicity remains (Juliano et al., 2018).

Additionally, Orellana et al. (2019) reported the use of nigericin, a novel, small molecule endosomal escape agent, to enhance the cytosolic delivery of folate-conjugated miRNA. Nigericin is a proton ionophore that exchanges osmotically inactive protons inside the endosomes with potassium ions in the cytosol. The combined high concentration of sodium and potassium ions raises the osmotic pressure inside the endosomes, resulting in endosomal rupture and release of the miRNA payload.

#### Intracellular Receptor Targeting as a Potential Endosomal Escape Strategy

For effective host cell infection, the Lassa virus (LASV, Jae et al., 2014) and ebolavirus (EBOV, Carette et al., 2011; Côté et al., 2011; Wang et al., 2016a) escape the endosome via a critical switch from their extracellular receptor (involved in cellular attachment and entry) to an intracellular endo-lysosomal receptor to mediate membrane fusion (Jae and Brummelkamp 2015). This is commonly due to the pH drop in the endosome that primes the viral glycoprotein (GP) for a receptor switch (Staring et al., 2018).

In particular, LASV was found to bind mainly to α-dystroglycan (Cao et al., 1998) as well as TAM receptor tyrosine kinases, DC-SIGN of dendritic cells, and C-type lectins of liver and lymph nodes (Shimojima et al., 2012) and is taken up mainly through macropinocytosis (Oppliger et al., 2016). The trimeric LASV spike protein is composed of a receptor-binding domain (GP1), a fusion protein subunit (GP2), and a unique stable signal peptide (SSP) (Burri et al., 2012) that directs the polypeptide to the endoplasmic reticulum and also interacts with GP2 during membrane fusion (Nunberg and York 2012). Structural studies support an entry model wherein endo-lysosomal pH (5.0–6.0) induces a conformational change in GP1 that facilitates an intracellular receptor switch to LAMP1, a late endosomal/lysosomal protein (Cohen-Dvashi et al., 2015; Li et al., 2016). Further acidification in the lysosomes (pH 4.0) sheds GP1, exposing GP2 that mediates membrane fusion (Li et al., 2016). The pH-dependence of the conformational change is attributed to the pH-sensing histidine triad on the surface of the spike protein (Cohen-Dvashi et al., 2015; Cohen-Dvashi et al., 2016). Mutation of these His residues reveals that LAMP1 binding is not necessary for membrane fusion but greatly enhances viral infection efficiency (Cohen-Dvashi et al., 2016).

Similarly, attachment of EBOV to the host cell membrane facilitates internalization principally through macropinocytosis (Nanbo et al., 2010), with evidence that the virus is also taken up via clathrin-mediated endocytosis (Aleksandrowicz et al., 2011). Several cell membrane contact sites have been identified that seem to facilitate virus attachment such as β1-integrins and Tyro3 (TAM) family kinase receptors, but no sites for direct interaction with the EBOV GP have been identified yet. C-type lectins (L-SIGN, DC-SIGN, and hMGL) have also been shown to enhance adherence of the virus to the host cell membrane. Due to the broad tropism of EBOV across different cell types and different host organisms, it has been difficult to identify cell surface receptors that facilitate internalization (Hunt et al., 2012). So far, TIM-1 was determined to be the EBOV receptor for epithelial cells (Kondratowicz et al., 2011). Upon entry, endo-lysosomal acidification activates proteases cathepsin B and cathepsin L that cleave the EBOV GP. Proteolysis reveals the active conformer GP2, which then binds to Niemann-Pick C1 (NPC1), a cholesterol transporter embedded on the endo-lysosomal membrane. This interaction facilitates the fusion of the viral and lysosomal membranes, releasing the viral nucleocapsid into the cytosol (Carette et al., 2011).

Because NPC1 is involved in vesicular trafficking, it is even more interesting that it is responsible for limiting lipid nanoparticle-mediated siRNA delivery by shuttling the bulk of the lipid nanoparticles back to the outside of the cell after endocytosis (Sahay et al., 2013). Moreover, inhibition of NPC1 greatly increases the cytosolic delivery of the siRNA cargo (Wang et al., 2016b). A similar effect was observed when ESCRT-1, another endo-lysosomal protein involved in vesicular sorting, was knocked down to enhance the delivery of a therapeutic anti-miRNA (Wagenaar et al., 2015). Alternatively, the entrapment of oligonucleotides in the late endosomes can be exploited. Instead of inhibiting or knocking down endo-lysosomal-associated proteins such as NPC1, LAMP1, or ESCRT-1, a ligand that engages the intracellular receptor can be used to facilitate the cytosolic delivery of the cargo. This could potentially be applicable to lipid-based systems where membrane fusion precedes content release.

## Nuclear Delivery

Unlike cytoplasmic viruses, nuclear viruses (such as SV40, adenovirus, influenza virus and HIV) need to travel further in order to replicate themselves in the nucleus of the host cell. They must cross a total of three cell barriers to reach the nucleus—the plasma membrane, cytosol and the nuclear membrane. Thus, they have evolved to use their structural features along with cellular transport machinery to hijack the well-protected nuclear import process. The size, structure, and composition of the viral proteins determines the mechanism by which it enters the nucleus. The structure and surface properties of nuclear viruses are also different from cytoplasmic viruses as the capsid of these viruses needs to be intact when they are traversing through the highly crowded cytosol but should breakdown in the perinuclear area (Cohen et al., 2011; Kobiler et al., 2012).

The nucleus is the main regulator of intracellular functions such as gene activation, cell division and proliferation, metabolism and protein production. As such, it is also considered as the most important target to deliver intact therapeutic exogenous oligonucleotides to treat diseases at the genetic level (Faustino et al., 2007; Pouton et al., 2007). However, cytosolic trafficking is a critical bottleneck for the efficient nuclear delivery of nucleic acids (Ni et al., 2019). Previous studies show that when a plasmid DNA is microinjected into the cytoplasm, the cellular enzymes degrade the DNA before it can reach the nucleus through Brownian motion (Cohen et al., 2009). Thus, it is necessary to protect as well as actively traffic the DNA to the perinuclear region.

To reach the nucleus, a number of different cytosolic trafficking strategies have been explored by nuclear viruses. Among these, the karyopherin-dependent and microtubule-assisted pathways have been extensively studied and mimicked for nucleic acid delivery (Bai et al., 2017). Thus, this section discusses these two common viral nuclear import mechanisms and how these pathways have inspired the development of nonviral vectors for therapeutic and diagnostic purposes (Cohen et al., 2011; Kobiler et al., 2012).

### Karyopherin-Mediated Pathway

The nuclear trafficking of the viral ribonucleoproteins (vRNPs) is required for production and release of mature virions. To travel actively toward the nucleus, viruses use nuclear localization signals (NLSs) to mediate nucleus entry of the vRNPS. NLS sequences are short basic peptide motifs that are recognized by karyopherin proteins and are transported to the nucleus via karyopherin α/β-mediated pathway (Cros and Palese 2003). Detailed chemical and biophysical studies show that the influenza A virus, herpes simplex virus, and SV40 consist of these NLS sequences embedded in their viral proteins. These specific sequences interact with the α subunit of dimeric karyopherin α/β receptors with high specificity. The karyopherin α binding site classifies the type of NLS as either classical or nonclassical. The classical NLS (derived from SV40) binds to inner concave surface of the ARM domain of karyopherin α. On the other hand, nonclassical NLS are the viral peptides that bind specifically and exclusively to the minor groove of the karyopherin α (Xu et al., 2005). An example is the NLS obtained from influenza A virus Li G. et al. (2019). The trimeric karyopherin-NLS complex docks at the nuclear pore complex and is passaged across the nuclear envelope and released into the interior. This transport mechanism is based on nucleocytoplasmic gradient of the GTP bound form of Ran protein as the Ran-GTP/GDP ratio is high in the nucleus but low in the cytoplasm. This difference in concentration acts as the driving force to transport the trimeric complex inside the nucleus (Fay and Panté, 2015).

**FIGURE 5:**
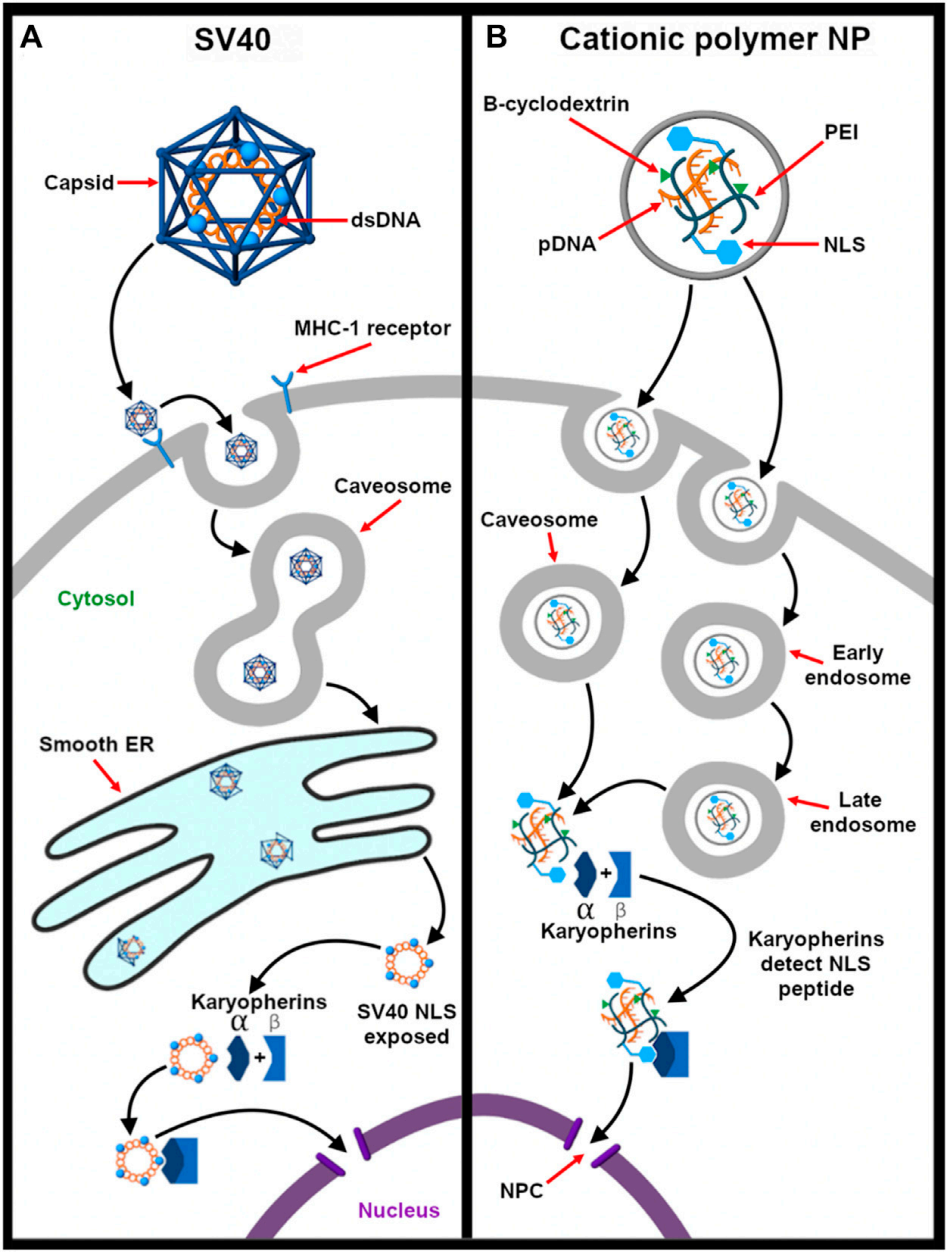
Karyopherin-mediated nuclear delivery of SV40 and of a synthetic nanovector. **(A)** SV40 binds to MHC-1 class receptors present on the host cell surface. This mediates the recruitment of caveolin-1 positive vesicles, and the virus is eventually taken up into caveosomes. These caveosomes undergo dynamic structural changes to form long tubular membrane extensions, which are then released from caveosomes and are transported to the smooth ER (Pelkmans et al., 2001). Once inside the ER lumen, the disassembly of viral capsid begins, and the partially disassembled capsid undergoes structural changes in the cytosol to expose the NLS embedded in the minor capsid. The NLS moiety is recognized by the karyopherin family, and the viral genome is transported to the nucleus as karyopherin cargo (Nakanishi et al., 2007; Toscano and de Haan 2018). **(B)** In this study by Hu et al. (2012), PEI conjugated to β-cyclodextrin (PC) was used to transfect pDNA. Results shows that it is internalized by caveolae- and clathrin-dependent pathways. To enhance the nuclear delivery of DNA, the NLS peptide inspired from SV40 virus was combined and conjugated to the PC backbone. Compared to PC/pDNA, PC/NLS/pDNA shows higher gene transfection efficiency.

Miller and Dean (2009) summarized nuclear targeting ligands that can be used to deliver therapeutic nucleic acids. These ligands can be easily modified and conjugated to the surface of a nanoparticle or directly to the gene of interest. Variants of virus-derived NLS peptides are most commonly used as nuclear targeting ligands (Kim et al., 2017). Thus, carriers decorated with or nucleic acid cargo associated with the NLS peptide sequence also undergo nuclear uptake via the karyopherin α/β pathway (Pan et al., 2012; Ray et al., 2015; Zanta et al., 1999; Cartier and Reszka, 2002). One such example by Hu et al., 2012 has been discussed in detail in Figure 5 wherein the classical NLS peptide sequence derived from SV40 virus was used to deliver a plasmid DNA (pDNA) polyplex across the nuclear envelope via karyopherin-dependent pathway (Hu et al., 2012). Importantly, the unmasking of NLS peptide in case of SV40 and HIV virus only when it is needed reduces the off-target binding and increases the karyopherin-mediated uptake (Nakanishi et al., 2002; Fanales-Belasio et al., 2010). These kinds of smart techniques can be explored further as current synthetic carriers are designed to deliver the whole construct to the nucleus and not just the nucleic acid cargo (Hu et al., 2012; Favaro et al., 2018).

Alternatively, the DNA nuclear-targeting sequence (DTS) is a 72 bp aptamer derived from SV40 and has innate affinity for NLS-tagged cytoplasmic proteins such as transcription factors (TFs) (Gaal et al., 2011). DTS-containing plasmids bind to one or more TFs, and the complex is shuttled into the nucleus. If cells are undergoing proliferation due to injury, the addition of DTS/NLS sequence shows limited effect in gene expression as the guard of the nuclear envelope breaks down (Miller and Dean, 2009). So far, DTS expressing plasmids have been delivered by electroporation or direct injection. Thus, it is possible to use DTS as a targeting ligand for gene vectors but not *in vivo*. In addition, plasmids complexed with proteins like HMG-1, histone H2B proteins, karyopherin receptors, and nucleoplasmin show increased transgene expression due to nuclear uptake (Miller and Dean, 2009).

### Microtubule-Assisted Transport

Many viruses use microtubule (MT) facilitated transport to traverse the cytoplasmic medium. Viral proteins induce rearrangement of microfilaments and recruit molecular motors such as dynein and kinesin to traverse from the plus to the minus terminal of MTs (Döhner et al., 2005). The MT-organizing center nucleates the minus end of the MTs and is close to the nucleus. This is how the viral capsid is transported actively to reach nearby regions of the nucleus (Naghavi and Walsh, 2017). Viruses such as the adenovirus, adeno-associated virus (AAV), and influenza A virus are able to hijack the cellular microtubule transport system, intercepting traffic to the nucleus. Amongst these, the adenovirus and influenza A virus are released out of the endosome before traveling along the microtubule in a non-vesicle dependent manner. In contrast, AAV is transported while within the endosome and the endosomal vesicle ruptures near the nucleus. The ligands that attach the endosomal membrane to the MT system are still currently unknown (Cohen et al., 2011).

**FIGURE 6:**
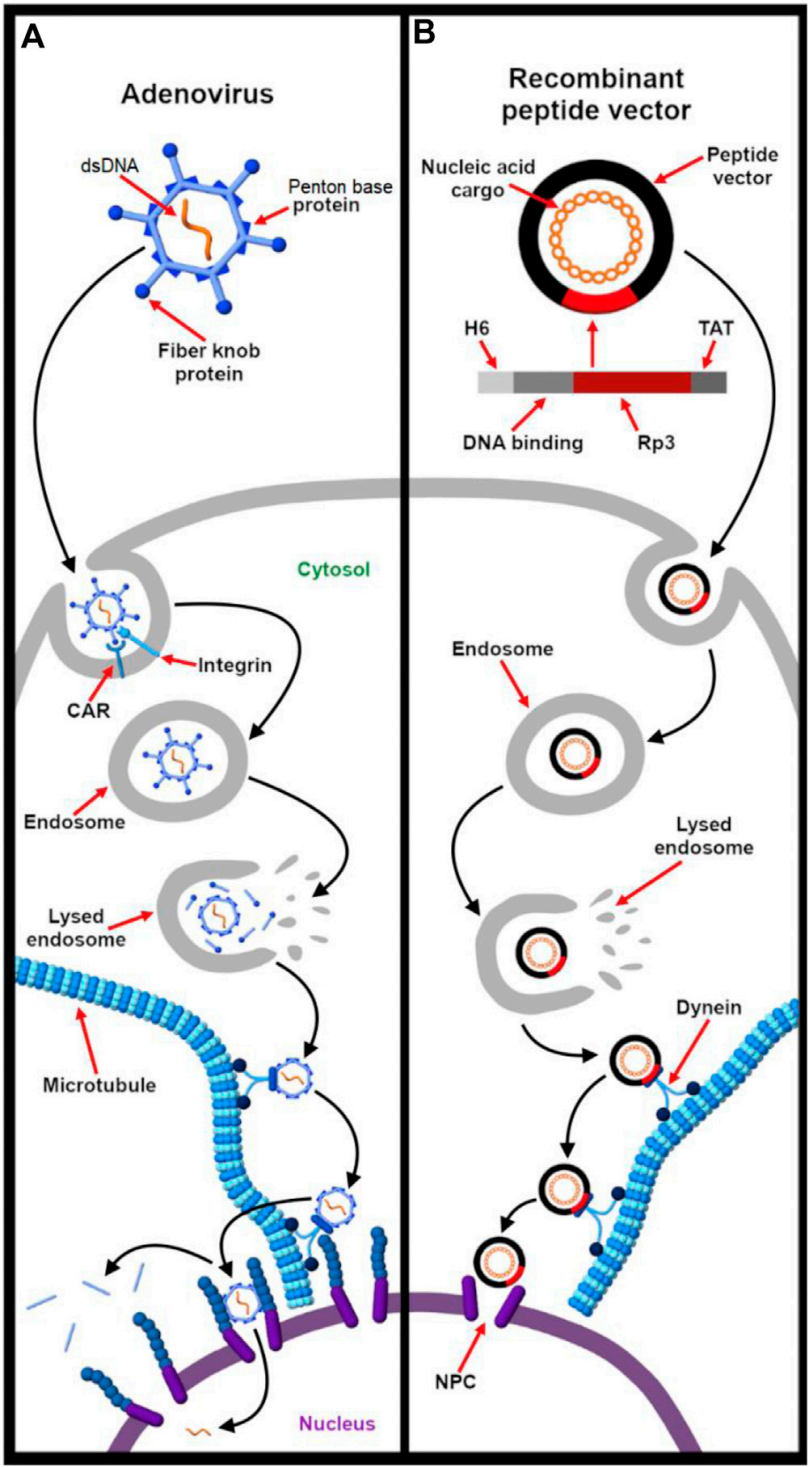
Microtubule (MT)-assisted nuclear delivery of adenovirus mimicked by a recombinant peptide vector. **(A)** Adenovirus undergoes receptor-mediated endocytosis by targeting CAR and integrin receptors present on the cell surface. Once inside the endosome, protein VI contains an N-terminal amphipathic helix that fragments the endosomal membrane. An adjacent peptide motif is also exposed which helps to drive the viral capsid out of the endosome (Flatt and Butcher 2019). After endosomal escape, the hexon facet of the viral capsid interacts with the kinesin light chain and cytoplasmic dynein protein. Thus, the virion hijacks the host’s dynein/dynactin motor proteins to hitchhike towards the nucleus. As the viral capsid docks onto the nuclear pore complex (NPC), the kinesin motor mediates a tug-of-war process for final uncoating of the viral capsid and release of the viral genome (Scherer and Vallee 2011). **(B)** To mimic this nuclear virus strategy, a peptide-based non-viral vector was synthesized by Favaro et al. (2018) wherein they used modular recombinant TRp3 protein (human dynein light chain) that interacts with dynein motor proteins and undergoes MT-assisted nuclear delivery. In addition to the MT-targeting protein, this peptide vector is composed of TAT for cell targeting, a DNA binding domain for electrostatic condensation of DNA and six histidine moieties for endosomal escape. Conclusively, this modular protein is able to efficiently deliver nucleic acid cargos including plasmid DNA, dsRNA and siRNA (Favaro et al., 2018).

In an effort to mimic viruses, the dynein binding protein (DBP) is often used as a ligand for nuclear uptake as it can mediate the transport of cargo via the MT-assisted pathway (Favaro et al., 2014; Favaro et al., 2018). A review by Midoux et al., 2017 has listed the dynein binding viral proteins and selective peptide sequences that have been used for efficient nonviral gene delivery. These peptides help to actively deliver the nanovector to the centrosome wherein the dynein interacts dynamically with the nuclear envelope and rearranges the nuclear lamin protein filaments, thereby increasing the permeability of nucleus (Dalmau-Mena et al., 2018). Moreover, Cohen and Granek (2014) provided theoretical insights on the rational design of spherical nanocarriers that require active transport to the nucleus. One recent example using such pathway is a peptide vector synthesized by Favaro et al., 2018 (2018). In this study, a dynein binding protein (TRp3) was incorporated into the vector to enhance microtubule-assisted delivery of an encapsulated gene toward the nucleus of the cell (Figure 6).

## Concluding Remarks

Evolution has honed viruses to be master hijackers of a broad range of host cells. They possess unique structural and mechanistic features wherein overarching themes such as capsid metastability, genome protection, stimuli-responsiveness, receptor duality, and synergistic ligand activity make them attractive templates for the design of non-viral nucleic acid carriers. Based on these outstanding characteristics of viruses, it is evident that an ideal carrier needs to find a balance between nucleic acid protection and release, two seemingly contradictory functions. A dynamic structure that responds to site-specific cues such as low pH or enzymatic activity help to control the release of nucleic acid cargo. These cues can vary with microenvironments within a cell, enabling biochemically controlled release mechanisms. Alternatively, the vector can be made sensitive to external stimuli such as light or temperature, which is more applicable to locally delivered formulations (Takemoto et al., 2014).

While therapeutic nucleic acids have made it to the clinical setting, extrahepatic targeting, endosomal escape, and controlled subcellular localization remain as major hurdles in their delivery (Juliano, 2016; Dowdy, 2017; Johannes and Lucchino, 2018; Juliano, 2018). Viruses commonly target multiple receptors for enhanced specificity and uptake, and this collective feature has been applied by synthetic carriers. Viral mimicry and the development of nucleic acid vectors iterate with our understanding of viral mechanism. Accordingly, advancements in techniques that identify viral ligands and corresponding host receptors, interrogate structure, and probe dynamics of ligand-receptor interactions may be translated to the design of more effective targeting domains for synthetic carriers.

In many ways, the outstanding difference in the transfection efficiency of viruses and synthetic vectors stems from the lack of a consensus of what drives endosomal escape. Escape from the endosome is influenced by a large range of factors such as nanoparticle properties (size, shape, and composition), mode of cellular uptake, and the type of cell (Selby et al., 2017). Moreover, mechanistic insights tend to be context-dependent as they are influenced by multiple factors such as the type of carrier, type of cell, and experimental conditions (LeCher et al., 2017). Structural studies on determinants of endosomal escape, while informative, often do not address the possible interplay of uptake route and intracellular trafficking. Moreover, uptake mechanisms are overlapping and poorly understood, making it difficult to determine the exact uptake mechanism of a particular construct (Nelemans and Gurevich, 2020). As uptake mechanisms typically involve signaling cascades, their relationship with intracellular trafficking are important considerations. Also, the implication of recycling pathways in viral and non-viral cytosolic access (Carette et al., 2011; Sahay et al., 2013; Wang et al., 2016a; Staring et al., 2018) suggests further studies on their exact role in therapeutic delivery. Filling such scientific gaps may help guide the design of more efficient nucleic acid delivery systems. Additionally, some viruses (such as the adenovirus) have been found to exploit cellular responses to membrane disruption concurrent with membrane fusion or penetration (Staring et al., 2018). In this light, future synthetic carriers may also be tailored to utilize host damage control to enhance therapeutic delivery. For this to be an effective strategy, it is imperative that the sensing of and response to invading particles by the host cell be exhaustively studied.

In summary, viruses can serve as a source of inspiration for chemists and materials scientists alike in the design considerations of non-viral vectors due to their efficient uptake and delivery of nucleic acid cargo. By designing nanoscale materials with stimuli-responsive properties and efficient targeting and internalization, therapeutic nucleic acids can be more rapidly brought forward for clinical application.
