# Corrigendum: Viral mimicry as a design template for nucleic acid nanocarriers

**DOI:** 10.3389/fchem.2025.1624509

**Published:** 2025-05-20

**Authors:** Ina F. de la Fuente, Shraddha S. Sawant, Mark Q. Tolentino, Patrick M. Corrigan, Jessica L. Rouge

**Affiliations:** Department of Chemistry, University of Connecticut, Storrs, CT, United States

**Keywords:** nucleic acid delivery, viral mimicry, endosomal escape, nuclear targeting, nanoparticles

In the published article, the **Funding** statement was omitted. The correct statement appears below:

“This work was supported by funding from the NIH under grant R35GM138226-02.”

The authors apologize for this error and state that this does not change the scientific conclusions of the article in any way. The original article has been updated.

